# Truncated Conjugate Structure Improves Solar‐Driven Hydrogen Peroxide Production Catalyzed by Benzobisthiazole‐Based Conjugated Polymers

**DOI:** 10.1002/advs.202518352

**Published:** 2025-11-23

**Authors:** Cui Li, Qirui Wang, Shu Lin, Xianglin Xiang, Kezhen Qi

**Affiliations:** ^1^ College of Pharmacy Dali University Dali Yunnan 671000 China; ^2^ Faculty of Materials Science and Engineering Kunming University of Science and Technology Kunming Yunnan 650093 China

**Keywords:** benzobisthiazole, conjugated polymers, hydrogen peroxide production, natural photocatalysis

## Abstract

The solar‐driven production of hydrogen peroxide from water and air presents a promising and sustainable alternative to the conventional anthraquinone oxidation process. In this work, a series of benzobisthiazole‐based conjugated polymers with tailored 1D, 2D, and 3D architectures are successfully constructed through precise dimensional engineering and molecular structure design. The study demonstrates that the 3D benzobisthiazole‐based conjugated polymer material (BBTz‐3D) possesses unique advantages due to its discontinuous conjugated structure. It can effectively utilize the truncation effect of sp^3^ C atoms in its molecular framework, which leads to the formation of localized electronic states within the material, thereby hindering the electron transfer pathway. Furthermore, the introduction of benzobisthiazole units expands the light absorption range of the system and facilitates the charge separation of photogenerated carriers. Therefore, the optimized system achieves a hydrogen peroxide generation rate of 7970.51 µmol g^−1^ h^−1^ in pure water. Moreover, under natural sunlight irradiation for three hours, the photocatalytic system demonstrated exceptional performance in Erhai Lake water, achieving a substantial hydrogen peroxide production of 6737.27 µmol g^−1^. This research presents a valuable approach for the rational design and synthesis of high‐performance organic photocatalysts, offering significant advancements in both fundamental understanding and practical applications of solar energy conversion.

## Graphical abstract

In this work, a novel molecular design strategy was employed to truncate large‐scale conjugated structures in covalent polymer materials. This approach significantly enhances the catalyst’s light‐harvesting capability, prolongs the lifetime of photogenerated charge carriers, and improves catalytic activity, thereby offering a new conceptual pathway for designing highly efficient photocatalysts.

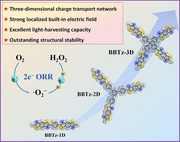

## 1 Introduction

Hydrogen peroxide (H_2_O_2_) has emerged as a significant raw material with diverse industrial applications and considerable potential as a renewable energy source. Its extensive applications encompass multiple sectors, including energy storage systems, environmental remediation processes, chemical manufacturing industries, and electronics production technologies.^[^ ^1^, ^2^, ^3^ ^]^ This widespread utilization has driven a substantial and rapid increase in global demand for hydrogen peroxide. The photocatalytic oxygen reduction reaction (ORR, Equations (1), (2), (3), (4)) coupled with water oxidation reaction (WOR, Equations (4), (5), (6), (7), (8)) presents a promising and sustainable alternative to the conventional anthraquinone oxidation method for H_2_O_2_ production.^[^ ^4^ ^]^ These methods offer effective solutions to meet the growing requirements for sustainable energy development, mitigate pollution and explosion risks associated with the traditional anthraquinone process, and reduce transportation expenses.^[^ ^4^, ^5^ ^]^ Nevertheless, the high recombination rates of photoinduced carriers and the sluggish reaction kinetics pose significant challenges to enhancing the efficiency of photocatalytic H_2_O_2_ production.^[^ ^6^ ^]^ Currently, the main types of photocatalysts that can produce H_2_O_2_ using solar energy include inorganic semiconductors (e.g., metal oxides and metal sulfides),^[^ ^7^, ^8^ ^]^ graphite carbon nitride (g‐C_3_N_4_),^[^ ^9^ ^]^ metal‐organic frameworks (MOFs),^[^ ^10^ ^]^ and covalent organic frameworks (COFs).^[^ ^11^ ^]^ Designing and fabricating a photocatalyst with precise control to attain outstanding photocatalytic H_2_O_2_ production performance remains a significant challenge.

ORR pathways (pH = 0):

(1) $$O_{2}+2e^{-}+2H^{+}\to \;H_{2}O_{2}\;E=+0.68\;V$$

(2) $$O_{2}+e^{-}\;\to \;\cdot {O_{2}}^{-}\;E=-0.33\;V$$

(3) $${O_{2}}^{-}+e^{-}+2H^{+}\to \;H_{2}O_{2}\;E=+1.44\;V$$

(4) $$O_{2}+4e^{-}+4H^{+}\to \;2H_{2}O\;E=+1.23\;V$$

WOR pathways (pH = 0):^[^ ^12^, ^13^ ^]^

(5) $$2H_{2}O+2h^{+}\to \;H_{2}O_{2}+2H^{+}E=+1.76\;V$$

(6) $$H_{2}O+h^{+}\to \cdot \mathrm{OH}+H^{+}E=+2.73\;V$$

(7) $$\cdot \mathrm{OH}+\cdot \mathrm{OH}\;\to \;H_{2}O_{2}$$

(8) $$2H_{2}O+4h^{+}\to \;O_{2}+4H^{+}E=+1.23\;V$$

Among various photocatalytic materials, conjugated polymers have recently demonstrated promising applications in the photocatalytic synthesis of H_2_O_2_ due to their tunable molecular structures, strong light absorption capabilities, and designable band structures.^[^ ^14^, ^15^, ^16^ ^]^ Compared to inorganic semiconductors, conjugated polymers enable precise optimization of photogenerated electron‐hole pair generation and separation efficiency through molecular engineering of their backbone structures, functional groups, and electronic properties, thereby enhancing photocatalytic performance.^[^ ^17^, ^18^, ^19^ ^]^ The thiazole group, as an electron‐accepting unit, can enhance light absorption capacity and promote charge separation.^[^ ^20^ ^]^ Benzobisthiazole or thiazole‐based conjugated polymers exhibit strong self‐assembly capability and high electron mobility, with their unique structural characteristics making them widely applicable in organic photovoltaic devices.^[^ ^21^ ^]^ Consequently, conjugated polymer photocatalysts derived from benzobisthiazole and thiazole are expected to demonstrate exceptional photocatalytic performance.

This study successfully synthesized 1D (BBTz‐1D), 2D (BBTz‐2D), and 3D (BBTz‐3D) benzobisthiazole‐based conjugated polymers based on benzobisthiazole units through an aldehyde‐imine condensation reaction between sulfur‐ and nitrogen‐containing heterocyclic compounds and aromatic aldehyde derivatives, followed by spontaneous oxidative dehydrogenation driven by aromatic stabilization. These conjugated polymers were used to generate hydrogen peroxide through photocatalysis from pure water and air without the presence of electron donors. Among them, BBTz‐3D showed the highest H_2_O_2_ productivity of 7970.51 µmol g^−1^ h^−1^. The results show that BBTz‐3D utilizes the conjugated truncation effect induced by sp^3^ C atoms in its molecular framework, thus generating localized electronic states and disrupting the electron transfer channels in the conjugated structure, while constructing a 3D charge transport network, and inducing a strong localized built‐in electric field, which significantly prolongs the charge lifetime and facilitates the efficient separation of photogenerated electrons and holes, thus achieving a significant improvement in photocatalytic performance. Furthermore, the synergistic integration of benzobisthiazole units enhances the material's performance by broadening the light absorption range and facilitating photoinduced charge separation. This study not only presents high‐performance materials for the photocatalytic synthesis of H_2_O_2_, but also unveils the synergistic interactions between 3D discontinuous conjugated structures and functional units, opening a novel route for the design of multifunctional conjugated polymers.

## 2 Results and Discussion

**Figure 1:**
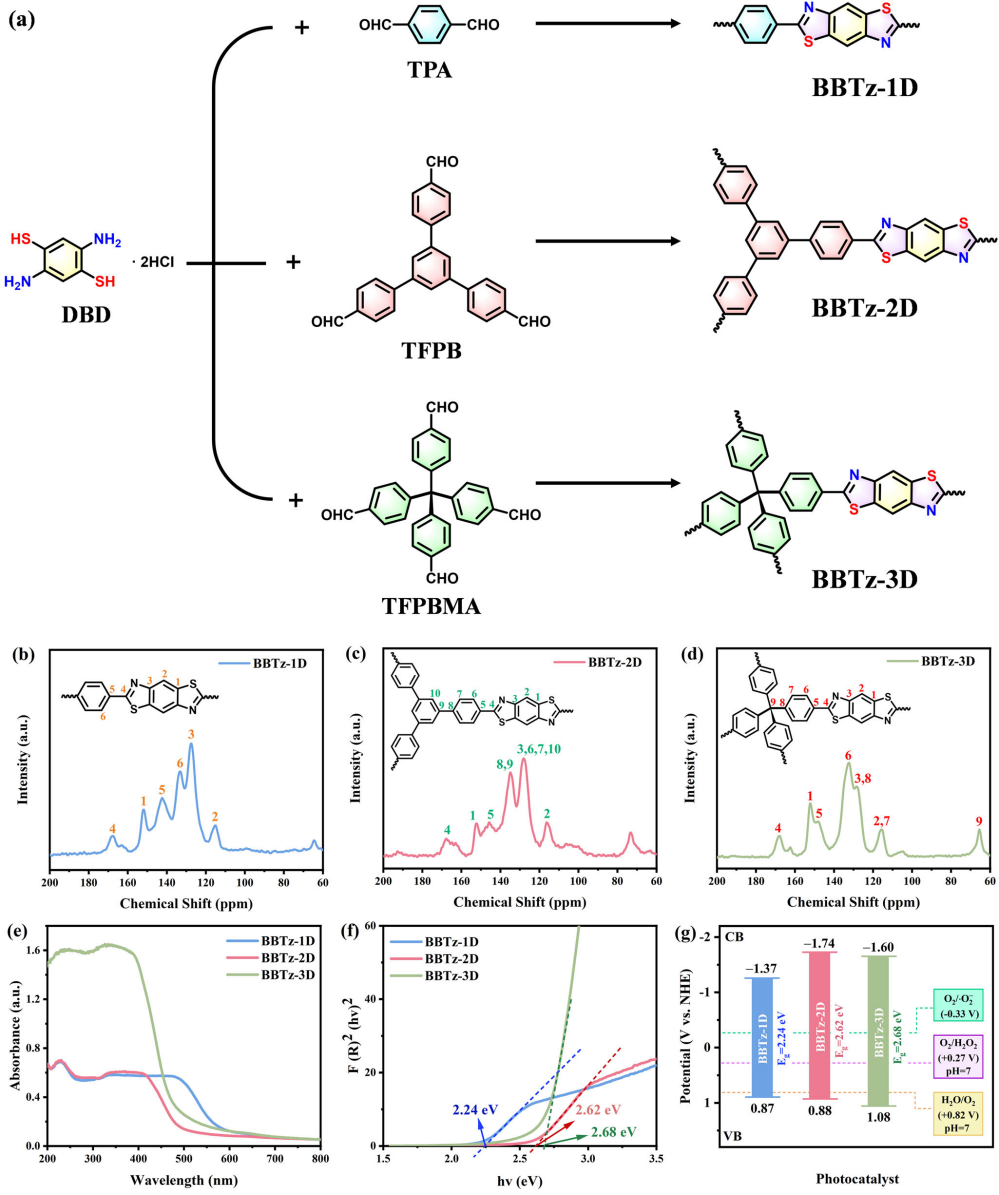
a) Synthetic route for BBTz–1D、BBTz–2D and BBTz–3D via aldimine condensation. Solid–state ^13^C–NMR spectra of the b) BBTz–1D, c) BBTz–2D and d) BBTz–3D. e) UV–vis diffuse reflectance spectra of BBTz–1D、BBTz–2D, and BBTz–3D. f) Tauc plots of the transformed Kubelka–Munk function vs. photon energy. g) Band alignment of benzobisthiazole–based conjugated polymers; the ORR and WOR related potentials were transformed to those at pH 7.

As shown in **Figure** **1**a, using N,N‐dimethylformamide (DMF) as the solvent, terephthalaldehyde (TPA), 1,3,5‐Tris(4‐formylphenyl)benzene (TFPB), and 4‐[tris(4‐formylphenyl)methyl]benzaldehyde (TFPBMA) underwent Schiff base reactions with 2,5‐Diaminobenzene‐1,4‐dithiol dihydrochloride (DBD). This process involved condensation between amino and aldehyde groups to form imine (C ═ N) bonds, followed by spontaneous oxidative dehydrogenation driven by aromatic stabilization, ultimately yielding benzobisthiazole‐based conjugated polymers: BBTz‐1D, BBTz‐2D, and BBTz‐3D. Detailed descriptions of the material synthesis procedures are provided in the Supporting Information. The structure of the sample was further characterized by Fourier transform infrared (FT‐IR) and solid‐state ^13^C nuclear magnetic resonance (^13^C‐SSNMR) spectra. The peaks of the Fourier transform infrared spectrum in the range of 1596–1599 cm^−1^ and 821–842 cm^−1^ can be attributed to the vibration of C ═ N and C─S in thiazole ring, respectively (Figure S2, Supporting Information). As shown in Figure 1b–d, the solid‐state ^13^C‐SSNMR spectra of all three conjugated polymers show a broad peak at ≈162 ppm, which can be attributed to thiazole carbon (S─C ═ N), indicating the formation of benzobisthiazole structure. Two peaks located at ≈152 and ≈142 ppm correspond to carbon atoms within the thiazole ring. The peak at ≈128 ppm can be assigned to the aromatic carbon atoms of benzobisthiazole.^[^ ^22^, ^23^, ^24^, ^25^ ^]^ This further confirmed the successful formation of the thiazole structure in benzobisthiazole‐based conjugated polymers.

X‐ray photoelectron spectroscopy (XPS) spectra (Figures S3–S5, Supporting Information) clearly show that C 1s, N 1s and S 2p signals are present in the skeleton structures of all three benzobisthiazole‐based conjugated polymers. The binding energy characteristics are highly consistent with the chemical environment of C─S, C ═ N and C ═ N─C/N─C─S in benzobisthiazole units, further confirming the successful construction of the thiazole structure in benzobisthiazole‐based conjugated polymers skeleton.^[^ ^24^, ^25^ ^]^ The binding energy shifts observed for benzobisthiazole‐based conjugated polymers with different dimensionalities reflect the regulatory effect of dimensional variation on local electronic structure. All the experimental results obtained from XPS analysis are highly consistent with the previous ^13^C‐SSNMR and FT‐IR analysis, confirming the successful formation of benzobisthiazole in the synthesized benzobisthiazole‐based conjugated polymers.

To investigate the porosity of benzobisthiazole‐based conjugated polymers, N_2_ adsorption isotherms were performed at 77 K. BBTz‐1D exhibits typical type‐III isotherms (Figure S6a, Supporting Information).^[^ ^26^ ^]^ The N_2_ adsorption–desorption isotherms of BBTz‐2D (Figure S6b, Supporting Information) and BBTz‐3D (Figure S6c, Supporting Information) showed the typical type‐IV isotherm, suggesting their mesoporous features.^[^ ^27^, ^28^ ^]^ The specific surface areas, as calculated by the Brunauer‐Emmett‐Teller method, for BBTz‐1D, BBTz‐2D, and BBTz‐3D are 34, 334, and 395 m^2^/g, respectively. The N_2_ adsorption–desorption isotherms indicated that the total pore volumes of BBTz‐1D, BBTz‐2D, and BBTz‐3D were 0.14, 0.32, and 0.40 cm^3^/g (P/P_0_ = 0.99), respectively. Thermogravimetric analysis (TGA) shows that all benzobisthiazole‐based conjugated polymers have excellent thermal stability in air atmosphere, and the decomposition temperature is higher than 580 °C (Figure S7, Supporting Information). X‐ray diffraction (XRD) shows that all photocatalysts are amorphous (Figure S8, Supporting Information), signifying typical conjugated polymers.^[^ ^16^, ^29^ ^]^

Figure S9 (Supporting Information) illustrates the surface morphology of the synthesized photocatalyst as observed by scanning electron microscopy (SEM). The structures of BBTz‐1D, BBTz‐2D, and BBTz‐3D all exhibit a densely packed, fused‐spherical configuration, showing an aggregated state. This morphology is attributed to the *π*‐extended benzobisthiazole framework, formed by inserting benzene units into thiazole, which promotes the self‐assembly of molecules in parallel stacking mode during the synthesis process, leading to the formation of compact nano‐particle aggregates and improving the planarity and the ability of self‐assembly of rigid structures.^[^ ^25^ ^]^ The morphological characteristics of BBTz‐1D (Figure S10a,b, Supporting Information), BBTz‐2D (Figure S11a,b, Supporting Information) and BBTz‐3D (Figure S12a,b, Supporting Information) were further verified by transmission electron microscope (TEM). The element mapping of energy dispersive X‐ray spectrum (EDS) shows that C, N and S are uniformly distributed in all samples (Figures S10c–f, S11c–f and S12c–f, Supporting Information).

In addition, the photoelectric characteristics of the synthesized benzobisthiazole‐based conjugated polymers were also studied. Solid‐state UV–vis diffuse reflectance spectroscopy (UV–vis DRS) shows that all the synthesized benzobisthiazole‐based conjugated polymers exhibit a wide absorption band (Figure 1e) between the ultraviolet and visible regions. According to the normalized UV–vis DRS in the figure, the absorption edge of BBTz‐1D, BBTz‐2D, and BBTz‐3D are 585, 500, and 488 nm, respectively, and gradually blue‐shift. Notably, the BBTz‐3D sample exhibits a significantly enhanced absorption efficiency, which can be attributed to photon scattering and multiple reflection effects within the 3D framework. The band gaps of BBTz‐1D, BBTz‐2D, and BBTz‐3D, estimated according to the Tauc diagram, are 2.24, 2.62, and 2.68 eV, respectively (Figure 1f). The linear long‐range continuous *π* conjugate structure of BBTz‐1D enhances the delocalization effect of electrons, resulting in a narrow band gap, and the absorption edge is located in the visible red region. In the 2D layered structure of BBTz‐2D, the *π* conjugate part in the plane interrupts the long‐range delocalization, which shortens the intramolecular conjugation. In addition, *π‐π* stacking between layers leads to exciton formation, and intermolecular charge transfer changes the electronic structure. Benzobisthiazole units in adjacent layers may form interlayer charge transfer through the weak interaction between thiazole sulfur atoms and benzene rings. The absorption energy level of this intermolecular charge transfer is higher than the intrinsic π‐π* transition, which leads to a blue‐shifted absorption toward the green region and an increased band gap. The sp^3^ C node in BBTz‐3D truncates *π*‐conjugation into short‐range domains, forming discrete localized electronic states, significantly increasing the band gap, and further blue shifting the absorption edge to the blue‐green region, with strong ultraviolet absorption. The color of BBTz‐1D, BBTz‐2D, and BBTz‐3D, ranging from orange‐red, yellow and light yellow, also reflects the change in the optical band gap (Figure S13, Supporting Information).

To gain a more precise understanding of the band positions, ultraviolet photoelectron spectroscopy (UPS) was utilized (Figure S14, Supporting Information). The valence band (VB) positions of BBTz‐1D, BBTz‐2D, and BBTz‐3D were calculated as 0.87, 0.88, and 1.08 V (vs. NHE), respectively, by subtracting the excitation energy of He I (21.22 eV) from the width of the UPS spectrum and subsequently adjusting for the vacuum energy level relative to the standard hydrogen electrode potential (4.44 eV). Their conduction band (CB) positions were determined to be −1.37, −1.74, and −1.60 V (vs. NHE), respectively. The energy band distribution of benzobisthiazole‐based conjugated polymers is shown in Figure 1g. The potentials for E(O_2_/H_2_O_2_) and E(H_2_O/O_2_) were converted to pH 7 conditions using the Nernst equation, yielding values of +0.27 V, and +0.82 V (vs. NHE), respectively. The O_2_/·O_2_^−^ reaction does not involve hydrogen ions (O_2_ + e^−^ = ·O_2_^−^), thus E(O_2_/·O_2_^−^) = −0.33 V (vs. NHE). Based on the band alignment analysis, the CB potentials of BBTz‐1D, BBTz‐2D, and BBTz‐3D are sufficiently negative to drive the two‐electron oxygen reduction reaction (2e^−^‐ORR). These results provide preliminary screening criteria for subsequent mechanistic investigations.

**Figure 2:**
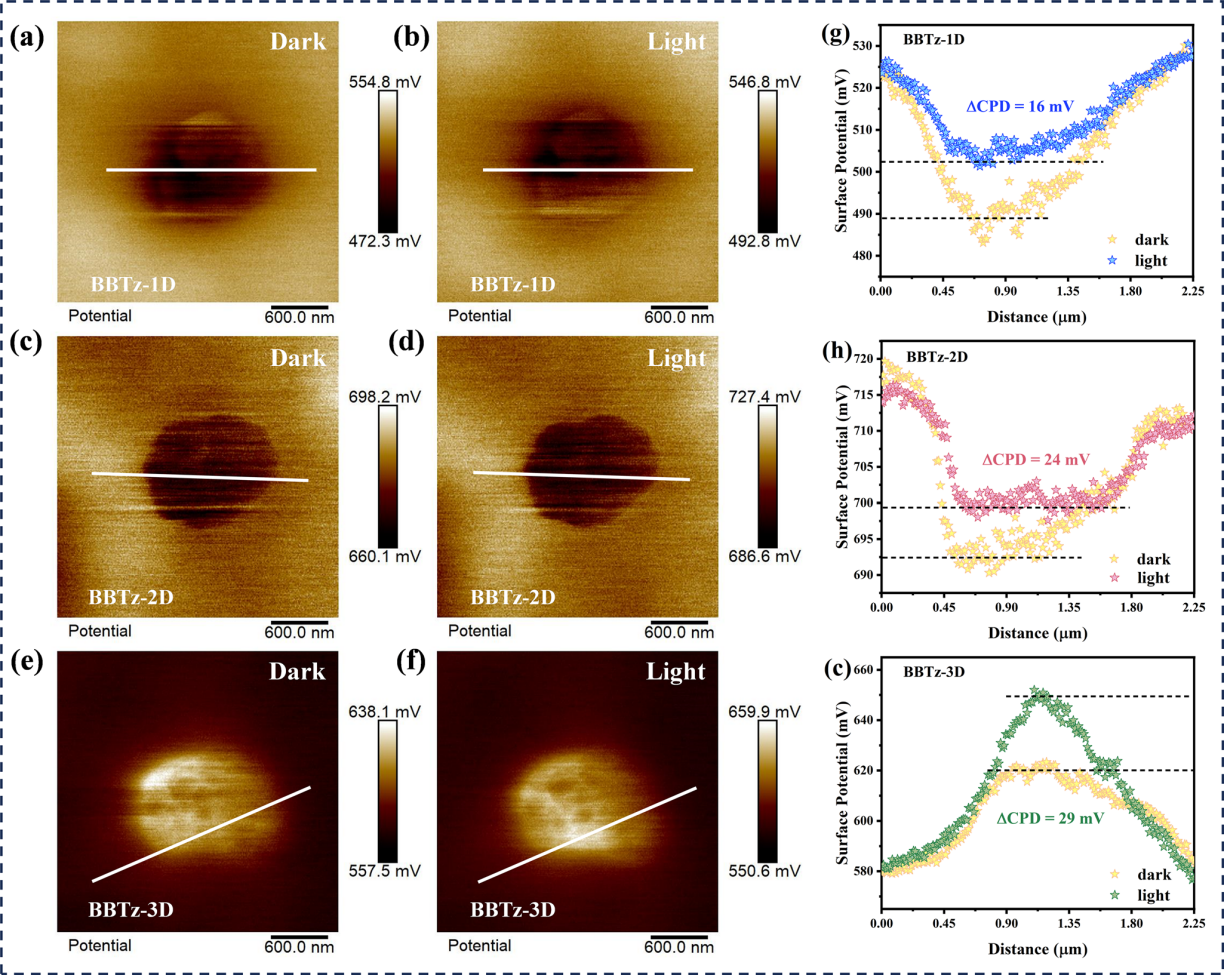
a,c,e) KPFM images of BBTz‐1D, BBTz‐2D, and BBTz‐3D under dark conditions. b,d,f) KPFM images of BBTz‐1D, BBTz‐2D, and BBTz‐3D under illumination conditions. g,h,i) The corresponding potential distribution curves at the marked lines.

To validate the process of photo‐induced charge separation on the surface of the sample, Kelvin probe force microscope (KPFM) measurement was employed to visualize the surface potential with nanoscale spatial resolution. It was noted that the potential difference for benzobisthiazole‐based conjugated polymers exhibited a positive increase under illumination, suggesting that the photogenerated holes migrated toward the surface (**Figure** **2**a–f).^[^ ^30^ ^]^ The ∆CPD (surface photovoltage, SPV) of BBTz‐1D, BBTz‐2D, and BBTz‐3D (Figure 2g–i) are 16, 24, and 29 mV, respectively. This indicates that BBTz‐3D possesses the most robust internal electric field, facilitating the directional movement of charges and enabling effective separation.^[^ ^31^ ^]^ The characteristics of the micro‐environment related to the charge within the photocatalyst were further examined using zeta potential measurements. Driven by the charge properties carried by the photocatalyst, the average zeta potentials recorded for the surfaces of BBTz‐1D, BBTz‐2D, and BBTz‐3D are +31.87, +25.03, and +17.23 mV, respectively, indicating a positive charge (Figure S15, Supporting Information). The linear chain configuration of BBTz‐1D results in a significant exposure of terminal functional groups, which enhances the density of positive charge on the surface. In contrast, while BBTz‐2D's layered structure minimizes edge defects, the weak interactions between its layers still result in the exposure of certain polar groups. The 2D cross‐linked architecture of BBTz‐3D facilitates a uniform distribution of charges across its discontinuous conjugate structure, leading to a reduced density of polar groups on the surface.

In addition, the electrochemical impedance spectroscopy (EIS), transient photocurrent (I‐t), fluorescence emission spectroscopy (PL) and time‐resolved fluorescence decay measurements were conducted to study the charge dynamics of the photocatalyst. The Nyquist plots reveal a systematic decrease in charge transfer resistance in the order: BBTz‐1D > BBTz‐2D > BBTz‐3D (Figure S16, Supporting Information). The EIS spectra of the three photocatalysts exhibit significant temperature dependence under different temperature conditions (Figure S17, Supporting Information). At low temperatures, the impedance values of the three materials are similar. As the temperature increases, the impedance of all materials decreases, which may be attributed to enhanced thermal vibrations and accelerated mass transfer processes within the electrochemical system. Notably, BBTz‐3D shows the most pronounced change, indicating that thermal vibrations play a critical role in promoting electron transfer across domains. As shown in Figure S18 (Supporting Information), consistent with the EIS results, BBTz‐3D shows the largest transient photocurrent response during multiple rounds of switching photocurrent testing. The excellent photoresponse of BBTz‐3D is the result of many factors, such as material structure, light absorption and photocurrent difference. The photocurrent response of benzobisthiazole‐based conjugated polymers shows a gradual decay process, which is caused by the rapid accumulation of surface charges on the sample under light excitation. The solid‐state emission peaks of BBTz‐1D, BBTz‐2D and BBTz‐3D are located at 590, 490, and 496 nm, respectively (Figure S19a, Supporting Information). The linear structure of BBTz‐1D provides continuous 1D conjugation pathways, enabling exciton delocalization along its continuous *π*‐conjugated chains.^[^ ^32^ ^]^ Meanwhile, the benzobisthiazole unit is a strong electron‐withdrawing group, and the 1D structure possesses a high density of benzobisthiazole units, which may lead to a stronger intramolecular charge transfer effect. Consequently, this results in a significantly red‐shifted emission peak for BBTz‐1D. Figure S19b (Supporting Information) depicts the fluorescence decay curves of the samples, and the average fluorescence lifetimes (τ_ave_) of BBTz‐1D, BBTz‐2D, and BBTz‐3D are 4.60, 4.95, and 6.03 ns (Table S1, Supporting Information), respectively. Although the continuous *π* conjugation of BBTz‐1D linear chain allows excitons to delocalize along the chain length, it is difficult for excitons to dissociate into free charges after delocalization due to the low built‐in electric field (ΔCPD = 16 mV) in 1D structure, which leads to a shortened fluorescence lifetime. The planar conjugation of the layered structure of BBTz‐2D provides moderate charge separation capability. The 3D discontinuous structure of BBTz‐3D is truncated and conjugated by sp^3^ C nodes to form localized electronic states, and at the same time, a 3D charge channel is constructed to improve carrier mobility. This spatial separation reduces the direct recombination of excitons and prolongs the fluorescence lifetime.

**Figure 3:**
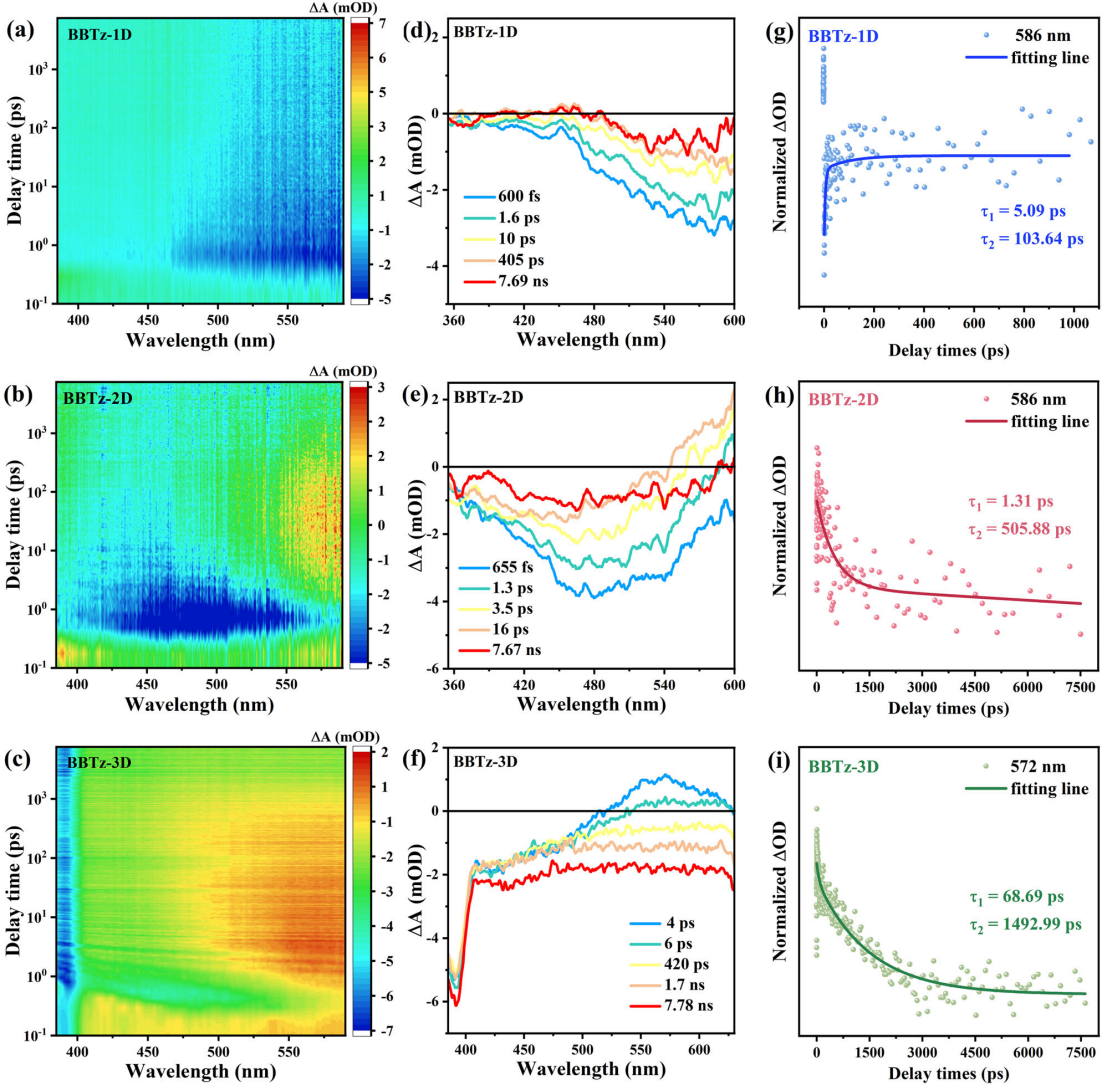
a–c) Pseudo‐color images of fs‐TA spectra signals on the fs‐ns timescales, d–f) fs‐TA spectra and g–i) fitted decay kinetic curves of BBTz‐1D, BBTz‐2D, and BBTz‐3D.

Studies have shown that the interface dipole caused by charge transfer can promote the dissociation of excitons into free carriers.^[^ ^33^ ^]^ To further understand the dynamic process of carrier separation and transfer, the excited state dynamics of BBTz‐1D, BBTz‐2D and BBTz‐3D were studied by femtosecond transient absorption (fs‐TA) spectroscopy. As shown in **Figure** **3**a–f, all the negative bleaching signals in the fs‐TA spectra, which can be attributed to the ground state bleaching (GSB) process, correspond to the generation of excited electrons.^[^ ^34^ ^]^ Notably, BBTz‐2D shows a weak and broad positive signal in the range of 540 nm to 600 nm, while BBTz‐3D exhibits a distinct positive absorption signal in the range of 500–630 nm, which can be attributed to excited state absorption (ESA).^[^ ^35^ ^]^ BBTz‐3D displays the strongest ESA absorption and the longest lifetime, confirming the most efficient separation of charge carriers. The results of kinetic fitting for benzobisthiazole‐based conjugated polymers indicate a biexponential decay process, characterized by a short lifetime (τ_1_) and a long lifetime (τ_2_). τ_1_ is associated with rapid exciton dissociation occurring at elevated exciton densities, whereas τ_2_ pertains to fast charge recombination and trapping phenomena (Figure 3g–i). Among them, the τ_1_ of BBTz‐2D is the smallest, reflecting its efficient exciton diffusion ability in the complete *π* plane, while the extended τ_1_ of BBTz‐3D may be due to the exciton cross‐domain hopping process caused by its discontinuous structure. Remarkably, the BBTz‐3D system exhibits substantially enhanced exciton dissociation efficiency compared to its lower‐dimensional counterparts (BBTz‐1D and BBTz‐2D), demonstrating that the 3D network compensates for the reduced single‐hop rate by increasing the number of dissociation interfaces. Furthermore, the prolonged τ_2_ lifetime suggests that the trapped long‐lived shallow electrons have a beneficial effect on active electron transfer, thereby contributing to enhanced photocatalytic performance.^[^ ^36^ ^]^ The charge separation efficiency of benzobisthiazole‐based conjugated polymers obtained from TA decay kinetics also follows the order of BBTz‐1D < BBTz‐2D < BBTz‐3D, which is consistent with the result obtained from the fluorescence lifetime decay curves.

**Figure 4:**
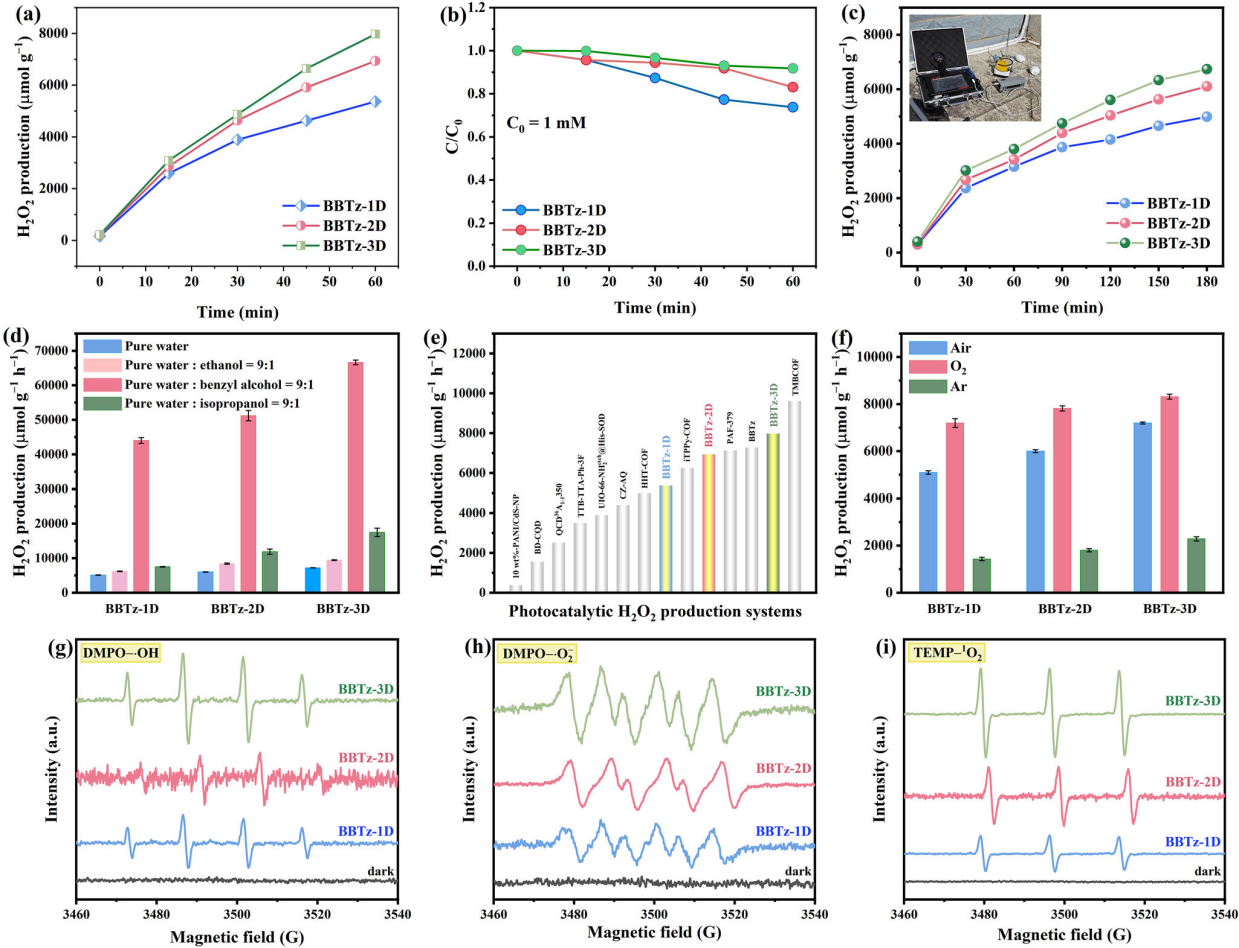
a) Typical time course of photocatalytic H_2_O_2_ production over different catalysts in pure water. b) H_2_O_2_ decomposition curves of BBTz‐1D, BBTz‐2D, and BBTz‐3D in pure water under light irradiation (C_0_ = 1 mm H_2_O_2_, N_2_ purging). c) Photocatalytic H_2_O_2_ production performance of benzobisthiazole‐based conjugated polymers in Erhai Lake water (Southwest China) under ambient atmospheric conditions. d) Photocatalytic H_2_O_2_ activity of the three benzobisthiazole‐based conjugated polymers in different reactive systems: pure water, pure water: ethanol = 9:1 (v/v), pure water: benzyl alcohol = 9:1 (v/v), and pure water: isopropyl alcohol = 9:1 (v/v). e) Comparison of BBTz‐1D, BBTz‐2D, and BBTz‐3D with representative materials in terms of the H_2_O_2_ production rate. f) The rate of H_2_O_2_ evolution catalyzed by BBTz‐1D, BBTz‐2D, and BBTz‐3D under different atmospheres. EPR trapping experiments of g) DMPO−·OH, h) DMPO−·O_2_^−^ and i) TEMP−^1^O_2_ on benzobisthiazole‐based conjugated polymers under light or dark conditions.

The photocatalytic H_2_O_2_ production performance of the prepared catalysts was evaluated under simulated solar irradiation using pure water as the reaction medium, without sacrificial agents or photosensitizers. This reaction system is cost‐effective and eco‐friendly, conforming to the concepts of sustainable development and green chemistry. Samples were taken at regular intervals, and H_2_O_2_ concentration was quantified via colorimetric analysis. As shown in **Figure** **4**a, the H_2_O_2_ production rates for BBTz‐1D, BBTz‐2D, and BBTz‐3D reached 5367.29, 6932.98, and 7970.51 µmol g^−1^ h^−1^, respectively, during the first hour of irradiation. When the reaction time was extended to 3 h, the H_2_O_2_ productivity of benzobisthiazole‐based conjugated polymers increased steadily, and the H_2_O_2_ productivity of BBTz‐1D, BBTz‐2D, and BBTz‐3D was 9034.85, 11900.80, and 12584.45 µmol g^−1^, respectively (Figure S20, Supporting Information). Since partial decomposition of H_2_O_2_ occurs during the actual photoreaction process, decomposition experiments were carried out in 1 mm H_2_O_2_ under an N_2_ atmosphere (Figure 4b). After 1 h of continuous irradiation, BBTz‐3D exhibited negligible H_2_O_2_ decomposition (< 10%), highlighting its significant potential for efficient photocatalytic H_2_O_2_ production. The exceptional performance of BBTz‐3D originates from its unique structural characteristics. The sp^3^ C node in BBTz‐3D increases the band gap moderately by cutting off the long‐range *π* conjugation, and forms localized electronic states in the material, blocking electron transfer channels in the conjugated structure. This localization effect not only significantly prolongs the fluorescence lifetime of photoinduced excitons and delays the recombination of photoinduced e^−^‐h^+^ pairs, but also promotes the directional migration of h^+^ to surface active sites through spatial localization, thus effectively quenching h^+^. Simultaneously, the strong built‐in electric field induced by sp^3^ C nodes further accelerates the carrier migration, drives the efficient separation and spatial decoupling of e^−^‐h^+^, and cooperates with the excellent light absorption ability of benzothiazole unit, realizes a remarkable improvement in photocatalytic H_2_O_2_ production performance. The apparent quantum yields (AQYs) of BBTz‐3D (0.05 g/L) reached 0.42% at 405 nm. Notably, AQY values exhibited a positive correlation with catalyst loading, achieving 0.74% at 30 mg loading (Figure S21, Supporting Information). A higher catalyst concentration enhances the density of active sites within the optical pathway, leading to improved photon absorption efficiency at 405 nm.

Furthermore, the H_2_O_2_ production efficiency was evaluated under white LED irradiation (770 nm ≥ λ ≥ 380 nm). Within 3 h, the H_2_O_2_ production of all photocatalysts was lower than that under xenon‐lamp irradiation, but the output was still not less than 6000 µmol g^−1^, indicating that the prepared benzobisthiazole‐based conjugated polymers exhibited excellent H_2_O_2_ production performance (Figure S22, Supporting Information). However, the trend of H_2_O_2_ production under white LED lamps was BBTz‐1D > BBTz‐2D > BBTz‐3D, which can be attributed to the different light absorption ranges of the materials.

Natural water bodies typically contain various dissolved ions and organic compounds, which can impede H_2_O_2_ formation by scavenging essential reactive radical intermediates.^[^ ^37^, ^38^ ^]^ In the photocatalytic system based on benzobisthiazole‐based conjugated polymers, real water samples with various water matrix conditions were used to verify their efficient H_2_O_2_ photocatalytic synthesis. The actual test shows that the total organic carbon (TOC) content in Erhai water is 6.944 mg/L, representing a typical natural water composition. Under natural conditions (3‐h exposure, Erhai Lake water, pH = 7.95), this system achieved remarkable H_2_O_2_ production amounts on BBTz‐1D, BBTz‐2D, and BBTz‐3D, which were 4989.28, 6104.56, and 6737.27 µmol g^−1^, respectively (Figure 4c). These results confirm the materials' capability for direct H_2_O_2_ photosynthesis across diverse water sources. In addition, the photocatalytic H_2_O_2_ production performance of BBTz‐3D was investigated under real outdoor conditions to evaluate its long‐term stability and practical applicability. The outdoor testing campaign was carried out from July 10 to 22, 2025 at Dali University (Dali, Yunnan, China), with monitoring of ambient temperature and corresponding H_2_O_2_ production rates throughout the experimental period (10 mg BBTz‐3D photocatalyst + 250 mL solvent). The outdoor image of the self‐assembled reactor is shown in Figure S23 (Supporting Information). During a six‐day outdoor experiment conducted under natural sunlight irradiation, BBTz‐3D was dispersed in both pure water and 10% benzyl alcohol aqueous solution, respectively. The characteristic climatic variations in the Yunnan region caused transient reductions in H_2_O_2_ accumulation during overcast/rainy periods and nighttime hours. In the pure water system, H_2_O_2_ production reached 9.90 mmol g^−1^ on the second day, with subsequent yields fluctuating due to changing weather conditions, achieving a maximum of 10.04 mmol g^−1^ on the final day (Figure S24a, Supporting Information). Remarkably, the system employing benzyl alcohol as a sacrificial agent demonstrated a consistently increasing H_2_O_2_ production trend, culminating in a remarkable yield of 744.97 mmol g^−1^ by the sixth day (Figure S24b, Supporting Information). These results demonstrate that BBTz‐3D possesses excellent photocatalytic activity and long‐term stability, making it a promising candidate for practical solar‐driven H_2_O_2_ production applications. This outstanding performance surpasses that of many previously reported photocatalysts (Table S2, Supporting Information).

The photocatalytic H_2_O_2_ production performance can be significantly enhanced through the introduction of sacrificial electron donors, which facilitate the separation of photogenerated charge carriers and accelerate reaction kinetics. When 10% (v/v) ethanol (EtOH) or 10% (v/v) isopropanol (IPA) aqueous solution was employed as sacrificial agents, a moderate increase in H_2_O_2_ production was observed. Strikingly, the addition of 10% (v/v) benzyl alcohol resulted in an approximately eightfold enhancement in H_2_O_2_ yield (Figure 4d). Ethanol and isopropanol act as hole scavengers, consuming photoinduced holes through oxidation reactions (EtOH → CH_3_CHO + 2H⁺ + 2e^−^; IPA → CH_3_COCH_3_ + 2H⁺ + 2e^−^), thereby reducing electron‐hole recombination probability. Additionally, the H⁺ released from alcohol oxidation participates in the oxygen reduction reaction (O_2_ + 2e^−^ + 2H⁺ → H_2_O_2_), addressing the bottleneck of insufficient proton supply in pure water and promoting the 2e^−^‐ORR pathway. The remarkable enhancement observed in the benzyl alcohol/water system originates from an effective phase‐separation mechanism. In this biphasic system, the benzobisthiazole‐based conjugated polymer photocatalyst predominantly resides in the benzyl alcohol phase (organic phase), while the generated H_2_O_2_ is confined to the aqueous phase, thereby avoiding the catalytic degradation of H_2_O_2_ by the conjugated polymer material. The biphasic water/benzyl alcohol system, leveraging phase separation, enables efficient H_2_O_2_ production. The generated H_2_O_2_ preferentially dissolves in the aqueous phase (pure water), while the benzobisthiazole‐based conjugated polymer photocatalyst remains confined to the organic phase (benzyl alcohol), thereby minimizing H_2_O_2_ decomposition and enhancing photocatalytic performance.^[^ ^39^, ^40^, ^41^ ^]^ Furthermore, the biphasic water/benzyl alcohol system establishes benzaldehyde‐mediated autocatalytic characteristics, which promote substantial accumulation of the ·OOH key intermediate, thereby significantly enhancing H_2_O_2_ production.^[^ ^42^ ^]^ The phenyl ring in benzyl alcohol not only strengthens the adsorption capability on the catalyst surface but also modulates the reaction pathway through its distinctive electronic structure, creating a synergistic effect between benzyl alcohol oxidation and H_2_O_2_ generation.^[^ ^43^ ^]^ Benzyl alcohol acts as a mass transfer gating to reconfigure the interfacial reaction of the photocatalyst, thereby activating previously inaccessible catalytic sites and fully leveraging its high surface area and superior optoelectronic properties.^[^ ^44^ ^]^ Consequently, benzyl alcohol exhibits distinct advantages over other sacrificial agents.

Under pure water conditions, all three materials exhibited a significant decline in activity after three consecutive cycles (Figure S25a, Supporting Information). This decrease can be attributed to the strong adsorption of reaction intermediates or final products on the catalyst surface, which may block active sites and hinder desorption. The introduction of 10% benzyl alcohol resulted in remarkable improvements in both activity and stability for all materials (Figure S25b–d, Supporting Information). Benzyl alcohol acts as a hole‐sacrificial agent, being oxidized to consume photogenerated holes. This effectively suppresses electron‐hole recombination and promotes the 2e^−^‐ORR for H_2_O_2_ generation. Concurrently, benzyl alcohol oxidation facilitates the elimination of accumulated intermediates or byproducts from the catalyst surface, thereby regenerating the active sites, which is particularly evident during the second cycle following the initial reaction in pure water. Most importantly, benzyl alcohol and water may form a biphasic system or microheterogeneous environment, facilitating the transfer of generated H_2_O_2_ from the catalyst surface into the aqueous phase. This reduces the residence time of H_2_O_2_ on the catalyst and minimizes opportunities for further side reactions. BBTz‐3D demonstrated the best long‐term stability, with the smallest decay in production rate by the fifth cycle. The characteristic peaks in XRD and FT‐IR spectra of BBTz‐1D, BBTz‐2D, and BBTz‐3D before and after the reaction did not change significantly, indicating that the synthesized benzobisthiazole‐based conjugated polymers had good structural stability (Figures S26 and S27, Supporting Information). However, surface state alterations or minor structural damage that are difficult to detect by these techniques cannot be entirely ruled out. As summarized in Figure 4e and Table S3 (Supporting Information), the photocatalytic H_2_O_2_ production efficiencies of various benzobisthiazole‐based conjugated polymer materials were systematically evaluated. The benzobisthiazole‐based conjugated polymers demonstrate superior photocatalytic H_2_O_2_ production efficiency compared to most conventional semiconductor materials.

To elucidate the photocatalytic reaction pathway of benzobisthiazole‐based conjugated polymers for H_2_O_2_ production, a series of comparative experiments was conducted under varied atmospheric conditions (Ar/Air/O_2_, Figure 4f). Under oxygen‐saturated conditions, the benzobisthiazole‐based conjugated polymers exhibited optimal H_2_O_2_ production performance, reaching 7196.60, 7816.80, and 8315.46 µmol g^−1^ h^−1^ for BBTz‐1D, BBTz‐2D, and BBTz‐3D, respectively. Under illumination, photogenerated electrons participate in ORR, converting O_2_ to superoxide radicals (·O_2_^−^). Notably, the H_2_O_2_ release rate of benzobisthiazole‐based conjugated polymers gradually increases with the increase of oxygen concentration. High concentration O_2_ accelerates electron capture, reduces electron‐hole recombination and promotes H_2_O_2_ generation, confirming dissolved oxygen is one of the sources of H_2_O_2_ generation. A small amount of H_2_O_2_ production is observed in the system under an Ar atmosphere. However, the valence band maximum (≈1.08 V) of the benzobisthiazole‐based copolymer is thermodynamically insufficient to drive the complete water oxidation reaction. Therefore, under Ar conditions, a pathway may exist in the system where direct water oxidation by valence band holes generates O_2_ (2H_2_O + 4h⁺ → O_2_ + 4H⁺, +0.82 V vs. NHE, pH = 7), followed by the ORR to produce trace amounts of H_2_O_2_ (O_2_ + 2e^−^ + 2H⁺ → H_2_O_2_, E = +0.27 V vs. NHE, pH = 7).^[^ ^21^ ^]^ Additionally, although the system operates under an argon atmosphere, trace amounts of oxygen adsorbed within the intrinsic pore structure of the catalyst cannot be completely eliminated by purging, and these residual O_2_ molecules can serve as the substrate for the ORR pathway.^[^ ^45^ ^]^ Band structure analysis reveals that the CB positions of all benzobisthiazole‐based conjugated polymers are sufficiently negative relative to standard ORR potentials (+0.27 V vs. NHE for direct ORR, and −0.33 V vs. NHE for indirect ORR, at pH = 7), thermodynamically favoring the 2e^−^‐ORR pathway. Importantly, benzobisthiazole‐based conjugated polymers maintain competitive H_2_O_2_ production in air atmosphere, highlighting their practical advantage of utilizing atmospheric oxygen.

To elucidate the mechanism of photocatalytic H_2_O_2_ generation, electron paramagnetic resonance (EPR) measurements were employed. Using 5,5‐dimethyl‐1‐pyrroline N‐oxide (DMPO) as a trapping agent, distinct signals corresponding to hydroxyl radical (·OH) and ·O_2_^−^ were observed. Figure 4g exhibits a 1:2:2:1 quartet pattern, characteristic of DMPO−·OH adducts, confirming the presence of hydroxyl radicals. Due to its instability, the H_2_O_2_ produced in the catalytic system is easily decomposed, and ·OH is the important decomposition product.^[^ ^46^ ^]^ Moreover, it is important to note that DMPO−·O_2_^−^ exhibits instability in aqueous solutions, which causes it to quickly undergo disproportionation or transformation into DMPO−·OH. Consequently, the presence of DMPO−·OH in water cannot be directly interpreted as evidence for the generation of ·OH.^[^ ^47^ ^]^ Because of its instability, the H_2_O_2_ produced in the catalytic system is easily decomposed, and ·OH is an important decomposition product.^[^ ^48^ ^]^ Additionally, Figure 4h displays six peaks (4 main peaks and 2 secondary peaks), characteristic peaks of ·OOH/·O_2_^−^ species, further supporting the involvement of oxygen‐centered radicals in the reaction pathway.^[^ ^49^ ^]^ Under visible light irradiation, obvious singlet oxygen (^1^O_2_) signal can be detected by using 2,2,6,6‐tetramethylpiperidine (TEMP) as a trapping agent. As shown in Figure 4i, three peaks with equal height, are the characteristic peaks of ^1^O_2_, unambiguously confirming the formation of ^1^O_2_. Under the same conditions, the signal intensities of the three photocatalysts are obviously different. Under light irradiation, BBTz‐3D shows much higher EPR signal intensity than other benzobisthiazole‐based conjugated polymers, indicating that it has better charge separation efficiency. The stronger charge separation ability leads to more e^−^ and h⁺ participating in the generation of reactive oxygen species (ROS), thus obtaining the best photocatalytic H_2_O_2_ production performance. Among them, the signal of TEMP−^1^O_2_ can be attributed to the oxidation of ·O_2_^−^ in the presence of photogenerated h^+^ (·O_2_^−^ + h^+^ → ^1^O_2_). In addition, the active species was not detected under dark conditions, indicating that the reaction was completely driven by light, excluding thermal catalysis or a spontaneous reaction path. Therefore, even without any sacrificial agent, the prepared photocatalyst still has high photocatalytic performance.

**Figure 5:**
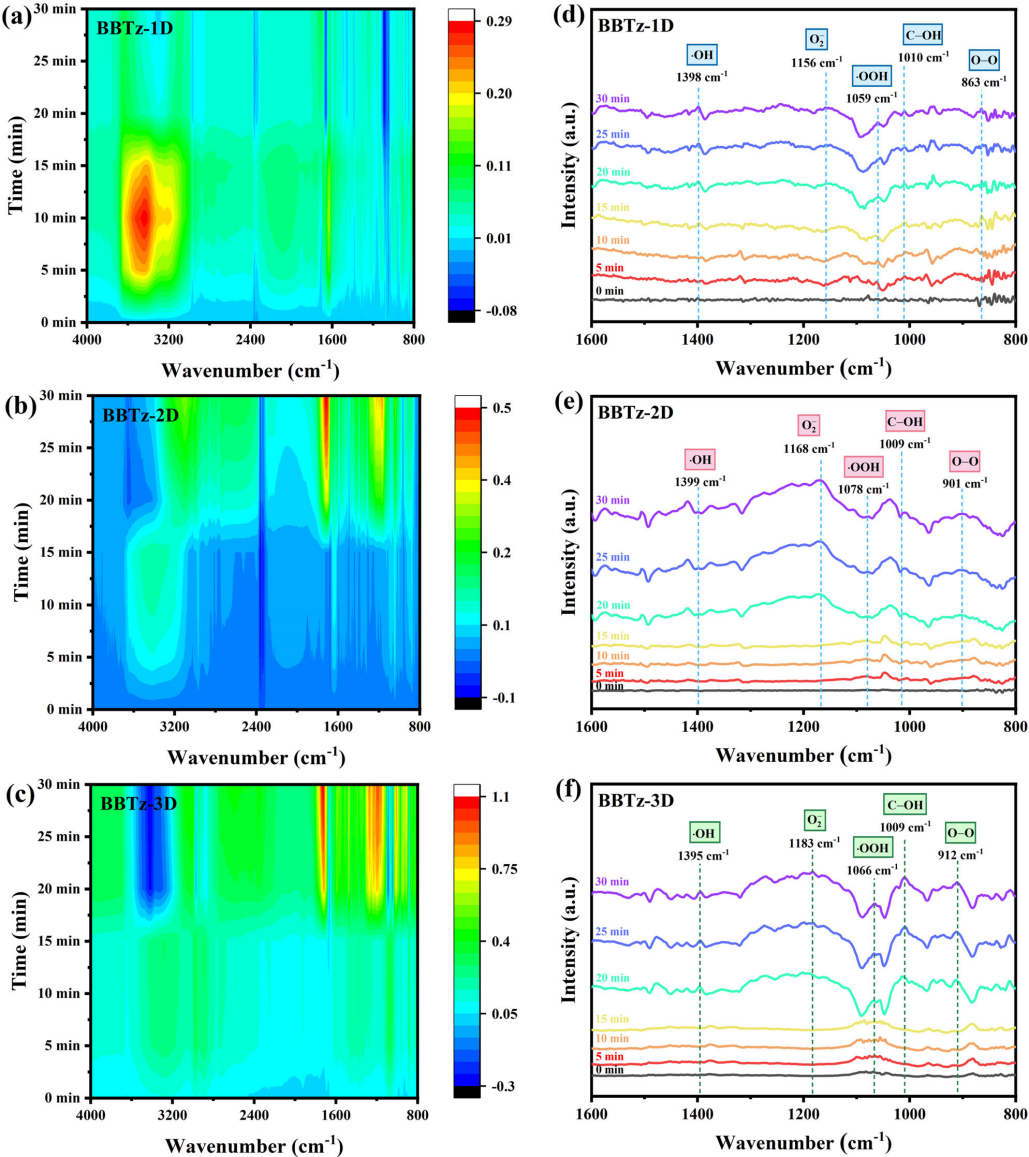
Pseudo‐color in situ DRIFTS plots of a) BBTz‐1D, b) BBTz‐2D, and c) BBTz‐3D under light irradiation. In situ DRIFTS spectra of d) BBTz‐1D, e) BBTz‐2D, and f) BBTz‐3D under illumination for 30 min at 5 min intervals.

In situ diffuse reflectance infrared Fourier transform spectroscopy (DRIFTS) was employed to further reveal the reaction intermediates in the photocatalytic process. As shown in **Figure** **5**, under illumination, the peak at ≈1010 cm^−1^ is attributed to the C−OH intermediate, indicating the formation of surface‐adsorbed *OH on benzobisthiazole‐based conjugated polymers. The signal intensities at ≈1183 cm^−1^ (·O_2_^−^) and ≈912 cm^−1^ (O−O) increased with the increase of illumination time, indicating that the adsorbed O_2_ (*O_2_) was reduced to ·O_2_^−^ by photogenerated electrons.^[^ ^48^, ^50^ ^]^ This is consistent with the observation results of EPR capture experiment. In addition, the signal at ≈1078 cm^−1^ proved the formation of *OOH species.^[^ ^51^ ^]^ These results further confirm that the photocatalytic mechanism of benzobisthiazole‐based conjugated polymers on H_2_O_2_ photosynthesis is the ORR pathway.

**Figure 6:**
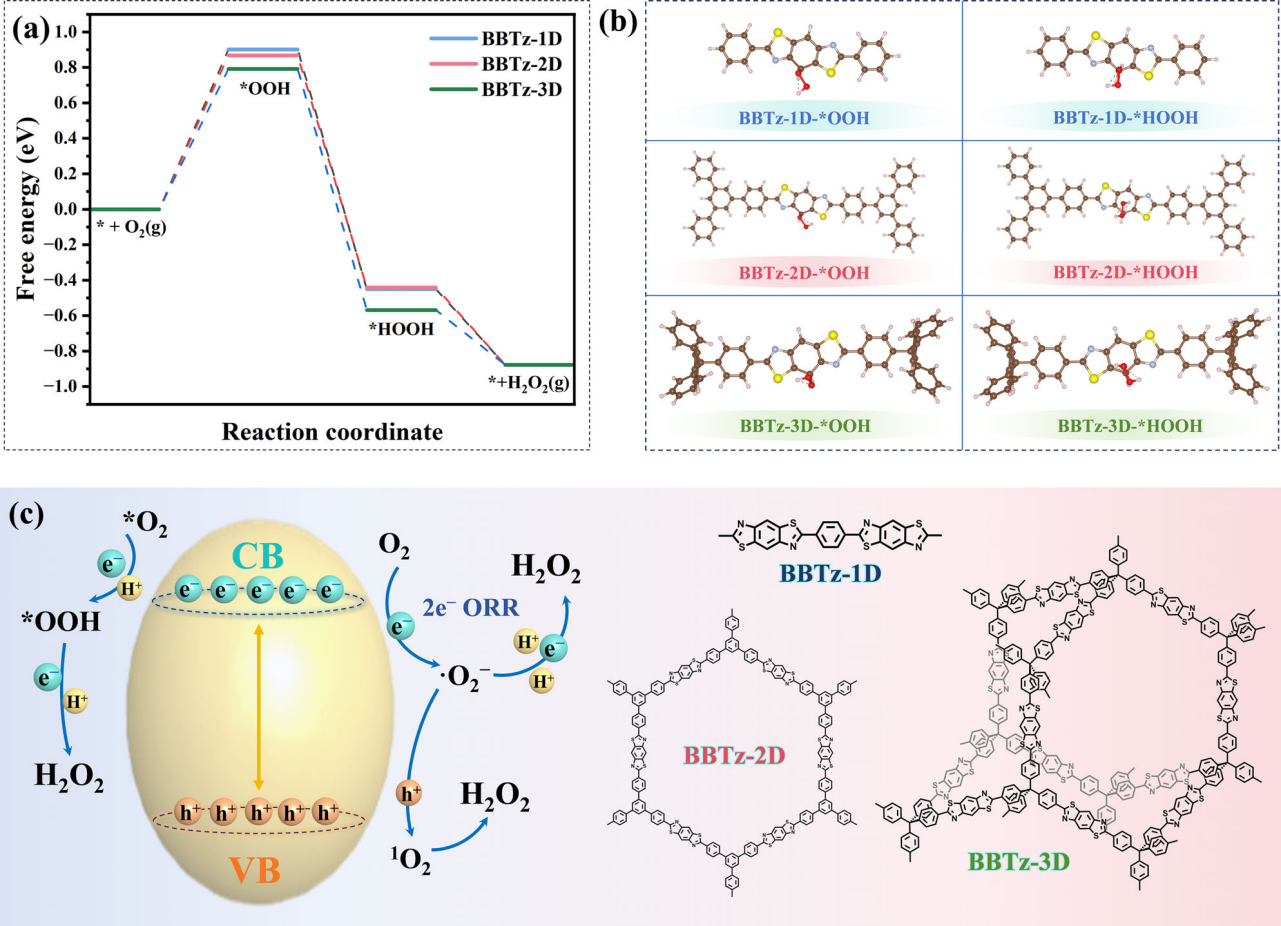
a) Calculated ΔG for the ORR pathways on benzobisthiazole‐based conjugated polymers. b) Different reaction intermediate of *OOH and *HOOH on BBTz‐1D, BBTz‐2D, and BBTz‐3D, respectively. Brown, pink, blue, and yellow spheres represent C, H, N, and S atoms, respectively. c) Proposed mechanism of photocatalytic H_2_O_2_ production over benzobisthiazole‐based conjugated polymers.

To comprehensively evaluate the photocatalytic performance of benzobisthiazole‐based conjugated polymers, we systematically investigated the Gibbs free energy changes (ΔG) along the 2e^−^‐ORR pathway through detailed density functional theory (DFT) simulations. This theoretical approach enables an understanding of the intrinsic correlation between the material's electronic structure characteristics and its catalytic reaction kinetics, thereby providing crucial insights into the structure‐activity relationships governing the photocatalytic process. The distribution patterns of the highest occupied molecular orbital (HOMO) and the lowest unoccupied molecular orbital (LUMO) orbitals for benzobisthiazole‐based conjugated polymers are shown in Figure S28 (Supporting Information). Within the benzobisthiazole‐based conjugated polymer fragment, a notable overlap is observed between the HOMO and LUMO, with both being delocalized throughout the entire conjugated framework. The electron density of the HOMO is mainly concentrated on the neighboring benzene ring units, whereas the LUMO is predominantly found on the benzobisthiazole unit. The benzobisthiazole may thus serve as active site for O_2_ adsorption and photocatalytic oxygen reduction.^[^ ^21^ ^]^ As shown in **Figure** **6**a, the benzothiazole site in benzobisthiazole‐based conjugated polymers shows a favorable Gibbs free energy change, indicating that O_2_ adsorbed on benzobisthiazole‐based conjugated polymers is more easily activated to form an *OOH intermediate. However, *OOH may be released from photocatalyst to form ·O_2_^−^, thus inhibiting 2e^−^‐ORR. In the presence of H^+^, *OOH intermediate can also be further reduced to form *HOOH (*OOH + H^+^ + e^−^ → H_2_O_2_) (Figure 6b). The energy barriers of BBTz‐1D, BBTz‐2D, and BBTz‐3D are 0.90, 0.86, and 0.79 eV, respectively. BBTz‐3D also shows the lowest formation energy from O_2_ to *OOH among all benzobisthiazole‐based conjugated polymers, indicating that it is more beneficial to 2e^−^‐ORR.^[^ ^52^ ^]^ This confirms that the order of H_2_O_2_ production by ORR follows the rules of BBTz‐3D > BBTz‐2D > BBTz‐1D, and efficient H_2_O_2_ production is achieved by one‐step 2e^−^‐ORR.

A computational model based on a tetrameric unit of benzobisthiazole was constructed, exhibiting an overall tetrahedral configuration. The benzobisthiazole units maintain coplanar conformations, indicating effective planar conjugation within individual segments. However, the connectivity between these units via sp^3^ C atoms disrupts continuous conjugation, consistent with the proposed conjugation truncation effect of sp^3^ C atom. Time‐Dependent Density Functional Theory (TD‐DFT) calculations were performed to simulate the UV–Vis absorption spectrum (Figure S29, Supporting Information). Some discrepancies were observed between the calculated spectrum of the model and the experimental optical absorption of the bulk material, which may be attributed to aggregation effects of the benzobisthiazole units in the solid state. Notably, a red shift in absorption was observed as the complexity of the model increased, reflecting the influence of extended intermolecular interactions. The macroscopic properties of organic functional materials are predominantly governed by their molecular packing and intermolecular interactions in the aggregated state. These interactions can significantly alter the electronic structure and excitation energies of the system, leading to substantial changes in spectral features and optical properties.^[^ ^53^, ^54^ ^]^ The system in this study, characterized by extended conjugation and strong intermolecular interactions, exhibits optical properties that are inherently dominated by its aggregated state structure. Molecular orbital diagrams of the BBTz‐3D tetramer model illustrate the HOMO and LUMO distributions for the S_0_→S_1_ (Figure S30, Supporting Information), S_0_→S_2_ (Figure S31, Supporting Information), and S_0_→S_3_ (Figure S32, Supporting Information) transitions. The analysis reveals that the transitions comprise a hybrid of localized *π*‐π* transitions within individual structural units and inter‐fragment charge transfer across units, indicating the occurrence of through‐space charge transfer upon photoexcitation in BBTz‐3D. Crucially, when the model was simplified to a two‐unit structure, such inter‐fragment charge transfer was not observed (Figures S33–S35, Supporting Information). Therefore, the phenomenon of inter‐fragment charge separation is an emergent property intrinsically associated with the unique 3D architecture of the BBTz‐3D material.

Combined with the above discussion, Figure 6c summarizes the reaction pathway mechanism of benzobisthiazole‐based conjugated polymers photocatalytic production of H_2_O_2_. The local electric field induced by the sp^3^ C node of BBTz‐3D and the charge transfer characteristics of benzobisthiazole reduce the adsorption energy of the intermediate, and produce H_2_O_2_ through the main route of 2e^−^‐ORR. In addition, the 3D conductive network drives h^+^ to oxidize H_2_O to generate O_2_ and H^+^, providing O_2_ and proton source for ORR. BBTz‐3D achieves a significant reduction in ORR free‐energy barrier and efficient utilization of photogenerated carriers through the cooperative design of discontinuous conjugate‐functional units, thereby achieving efficient photocatalytic H_2_O_2_ production.

## 3 Conclusion

In this study, a 3D benzobisthiazole‐based conjugated polymer material with efficient photocatalytic H_2_O_2_ production performance was successfully constructed by coupling the innovative 3D discontinuous conjugate structure design with the functional unit of benzobisthiazole. The optimized BBTz‐3D achieved a remarkable H_2_O_2_ production rate of 7970.51 µmol g^−1^ h^−1^ under illumination without electron donors. BBTz‐3D leverages the truncation effect of the conjugated structure of sp^3^ C atoms in the molecular structure to form localized electronic states in the material, blocking the transmission channels in the conjugated structure of electrons. This induces a strong localized built‐in electric field, significantly prolonging the charge carrier lifetimes, and realizing the efficient separation and directional migration of photogenerated electrons and holes, ultimately yielding superior photocatalytic H_2_O_2_ production performance. This study provides a new strategy for designing and synthesizing high‐performance photocatalysts for H_2_O_2_ photocatalytic production.

## Conflict of Interest

The authors declare that they have no known competing financial interests or personal relationships that could have appeared to influence the work reported in this paper.

## Supporting information



Supporting Information

## Data Availability

The data that support the findings of this study are available on request from the corresponding author. The data are not publicly available due to privacy or ethical restrictions.
