# Supplementary material for: Truncated Conjugate Structure Improves Solar‐Driven Hydrogen Peroxide Production Catalyzed by Benzobisthiazole‐Based Conjugated Polymers
Source: Adv Sci (Weinh). 2025 Nov 23;13(7):e18352. doi: 10.1002/advs.202518352 (PMC12866696; doi:10.1002/advs.202518352)
Supplement: Supplementary file 1 — Supporting Information [file ADVS-13-e18352-s001.docx]

Supplementary Information

**Truncated conjugate structure improves solar-driven hydrogen peroxide production catalyzed by benzobisthiazole-based conjugated polymers**

Cui Li^1^, Qirui Wang^1^, Shu Lin^1,*^, Xianglin Xiang^2,*^, Kezhen Qi^1,*^

1 College of Pharmacy, Dali University, Dali 671000, Yunnan, China

2 Faculty of Materials Science and Engineering, Kunming University of Science and Technology, Kunming 650093, Yunnan, China

1. **Experimental Section**

**1.1 Reagents and solvents**

All solvents and reagents were used without further purification. 2,5-Diaminobenzene-1,4-dithiol dihydrochloride (DBD, 97%), terephthalaldehyde (TPA, 98%), 1,3,5-Tris(4-formylphenyl)benzene (TFPB, 98%), and 4-[tris(4-formylphenyl)methyl]benzaldehyde (TFPBMA, 97%) were purchased from Zhengzhou Alpha Chemical Co., Ltd. N,N-dimethylformamide (DMF, 99.5%) was provided by Shanghai Macklin Biochemical Co., Ltd.

**1.2 Synthesis of** **benzobisthiazole-based conjugated polymers**

1D (BBTz-1D), 2D (BBTz-2D), and 3D (BBTz-3D) benzobisthiazole-based conjugated polymers were synthesized by reacting 1 mmol of TPA, 1 mmol of TFPB, and 1 mmol of TFPBMA with 1 mmol, 1.5 mmol, or 2 mmol of DBD respectively, in 10 mL of DMF. The reactions were carried out in a high-pressure reactor at 180 °C for 8 h. After cooling to room temperature, the products were washed with deionized water (3~6 times) and subsequently freeze-dried to obtain the final products.

**1.3 Characterization methods**

The crystal phase of the prepared samples was analyzed using a powder X-ray diffractometer (XRD, Bruker D8 Advance). The XRD measurements were performed with Cu-Kα radiation (λ = 1.5406 Å) under an accelerating voltage of 40 kV and a current of 30 mA. The surface chemical state of the catalyst was analyzed using an X-ray photoelectron spectrometer (XPS, Thermo Fisher ESCALAB 250). All binding energies were calibrated with reference to the C 1s spectral line at 284.8 eV. The specific surface area, pore volume, and pore size distribution of the catalyst were determined by low-temperature N_2_ adsorption-desorption measurements using a BSD-660M A3M analyzer. Prior to analysis, approximately 100 mg of the sample was degassed at 100°C for 2 h under vacuum. Then, the sample was put in a liquid nitrogen bath at −196.15 ºC (77 K) for testing. The specific surface area was calculated using the multipoint Brunauer-Emmett-Teller (BET) method, while the pore size distribution and average pore diameter were derived from the Barrett-Joyner-Halenda (BJH) model. Fourier transform infrared (FT-IR) spectra were recorded on a Thermo Scientific Nicolet 380 spectrometer. Additionally, the in situ diffuse reflectance infrared Fourier transform spectroscopy (DRIFTS) was performed using a Bruker IFS66V spectrometer equipped with a Harrick diffuse reflectance accessory. Solid-state ^13^C nuclear magnetic resonance (^13^C-SSNMR) were acquired on a Bruker BioSpin Avance III HD 400 MHz spectrometer. The thermal stability of synthesized materials was evaluated using a DZ-TGA105 thermogravimetric analyzer under a temperature range of 10~800 ºC. The Zeta potential analysis of catalyst was measured at 25 ºC using a Malvern Zetasizer. The surface morphology and physicochemical properties of the samples were examined using a scanning electron microscope (SEM, SM-7500F, JEOL). The microstructure, elemental distribution, and local composition of the samples were analyzed using a transmission electron microscope (TEM, JEM-2100F, JEOL) coupled with energy-dispersive X-ray spectroscopy (EDS) for elemental mapping. The contact potential difference (CPD) was measured using a Bruker Dimension Icon atomic force microscope in Kelvin probe force microscopy (KPFM) mode. The probe model is SCM-PIT, the scanning frequency is 1 Hz, and the scanning area is 34 μm^2^. The room-temperature photoluminescence spectrum (PL) of the prepared sample was measured by a RF-5301 (Shimadzu) fluorescence spectrophotometer. A UV-2600i (Shimadzu) ultraviolet-visible spectrophotometer equipped with an integrating sphere was used to measure the light absorption properties of solid samples. Prior to measurement, a BaSO_4_ baseline correction was performed in the range of 200~800 nm. The bandgap energy (E_g_) was estimated using the Tauc plot method based on the following equation:

$${(\alpha h\nu)}^{1/n}=A(h\nu-E_{g})$$

Where “α” is adsorption coefficient, “hν” is the photon energy, and “A” is a constant. The value of “n” is determined by the nature of electronic transition. Generally, “n” = 1/2 corresponds to a direct bandgap transition, while “n” = 2 indicates an indirect bandgap transition. In this work, given the direct transition property of benzobisthiazole-based conjugated polymer, the “n” value was assigned as 1/2.

Ultraviolet photoelectron spectroscopy (UPS) measurements were performed on a Thermo SCIENTIFIC Nexsa spectrometer using He I line (21.22 eV). During the measurements, a bias of −5 V was applied between the sample and the analyzer. The following equation should then hold:

$$\Phi=h\nu-\left( E_{\mathrm{cutoff}}-E_{\mathrm{fermi}} \right)$$

where Φ is the work function obtained from UPS, E_fermi_ is the value of Fermi edge from Fermi level (set at 0 eV) to the plot onset, and E_cutoff_ is the value of the tail cutoff at the low kinetic energy end of the plot, for a He I light source, hν = 21.22 eV. Φ is also equal to the valence band (VB) relative to vacuum. And 4.44 eV is the energy difference between NHE and the vacuum level. So, convert VB to NHE:

$$E_{VB, NHE}=E_{\mathrm{VB}}-4.44$$

The conduction band (CB) is calculated as follows:

$$E_{\mathrm{CB}}=E_{\mathrm{VB}}-E_{g}$$

The free radicals produced by photocatalyst before and after irradiation were qualitatively analyzed by Bruker EMX plus electron paramagnetic resonance (EPR). The trapping agents were 5,5-dimethyl-1-pyrroline-N-oxide (DMPO) and 2,2,6,6-tetramethylpiperidine (TEMP).

**1.4 Electrochemical measurements**

The electrochemical tests were carried out using a CHI-660D (CH Instruments) electrochemical workstation with a standard three-electrode configuration. The working electrode is indium tin oxide (ITO) conductive glass coated with the prepared catalyst, the reference electrode and the counter electrode are Ag/AgCl and platinum electrode respectively. 0.1 M Na_2_SO_4_ is used as the electrolytic solution. The catalyst slurry was prepared by ultrasonically dispersing 10 mg sample in a mixture of 1800 μL ethanol and 200 μL Nafion solution (5 wt%), followed by magnetic stirring for 4 hours. The homogeneous suspension was drop-cast onto pre-cleaned ITO substrates and air-dried before being mounted in the electrode holder.

**2 Computational details**

Calculations based on first-principles density functional theory (DFT), including molecular dynamics simulations were executed utilizing the Vienna Ab initio Simulation Package (VASP) ^[1]^ in conjunction with the Projector Augmented Wave (PAW) methodology.^[2]^ The exchange-correlation functional was managed within the parameters of the Generalized Gradient Approximation (GGA), adopting the Perdew-Burke-Ernzerhof (PBE) functional.^[3]^ The long-range van der Waals interactions are accounted for through the DFT-D3 approach.^[4]^ We implemented a plane wave basis set with an energy cutoff set at 500 eV, and the geometric relaxation was carried through until the forces acting on each atom were less than 0.03 eV/Å. The sampling of the Brillouin zone was conducted using a 1 × 1 × 1 k-point grid. To assure rigorous consistency, calculations were performed until the energy convergence threshold was less than 10^−5^ eV.

The free energy of the intermediates is calculated：

$$\Delta G=\Delta E_{\mathrm{DFT}}+\Delta ZPE-T\Delta S$$

where ΔE_DFT_, ΔZPE and ΔS are the changes of the reaction energy obtained from DFT calculations, zero-point energy, and the changes of entropy from the initial state to the final state, respectively. T is temperature and the T of 298.15 K was used in all computations.

The ground state (S_0_) was optimized using DFT combined with the M06-2X/def2-SVP^[5-7]^ level. The excitation energies were accurately calculated using the time-dependent density functional theory (TD-DFT) method combined with the M06-2X/def2-TZVP level. In order to account for matrix effects in the condensed phase and to allow a better comparison to available experimental data, all calculations were conducted using the SMD^[8-10]^ solvation model, taking the dielectric constant for water (ε = 78.39) as reference. The absence of imaginary frequencies in the optimized structures indicated that all the structures were at a local energy minimum. All the calculations described above were carried out with the Gaussian16^[11]^ code. IFCT^[12]^ and hole-electron method^[13]^ were used to analyze the electron excitation characteristics. These calculations were performed by Multiwfn^[12, 14]^, and visualized using VMD^[15]^.

**3 Photocatalytic experiment and H_2_O_2_ detection**

**3.1 H_2_O_2_ standard curve**

A series of hydrogen peroxide (H_2_O_2_) standard solutions with concentrations ranging from 0 to 600 μmol L^−1^ (in 100 μmol L^−1^ increments) were prepared for calibration. The generated H_2_O_2_ concentration was determined by iodometry based on the following redox reaction under acidic conditions. For spectrophotometric analysis, 1.0 mL aliquots of each standard solution were mixed with 1.0 mL of 0.4 M potassium iodide (98.5%) solution and 1.0 mL of 0.1 M potassium hydrogen phthalate (99.8%) solution. The reaction mixtures were then incubated at 30 °C in a water bath for 30 min under dark conditions. Subsequently, the absorbance of the obtained solutions was measured at 351 nm using an ultraviolet–visible spectrophotometer (Figure S1a). A standard calibration curve was generated by plotting the measured absorbance values against the corresponding H_2_O_2_ concentrations (Figure S1b).

$$H_{2}O_{2}+3I^{-}+2H^{+}\to I_{3}^{-}+H_{2}O$$


**Figure S1.** (a) Correlation between H_2_O_2_ concentration and UV-vis absorbance at 351 nm. (b) The standard calibration curve for H_2_O_2_ quantification.

**3.2 Experiment on photocatalytic production of H_2_O_2_**

The photocatalytic H_2_O_2_ production was carried out in a quartz reactor containing 5 mg photocatalyst, 100 mL pure water under the irradiation of a 300 W xenon lamp (CEL-HXF300-T3, CEAULIGHT) or a 24 W white LED (MC-LED20, MC Magnesium Rui Chen). After the reaction, 1 mL of the mixture was filtered through a 0.22 μm membranes to remove catalyst particles for the subsequent quantitative determination of H_2_O_2_.

**3.3 Photocatalytic decomposition of H_2_O_2_**

The photocatalytic decomposition experiment was conducted as follows: 5 mg of the photocatalyst was dispersed in 100 mL of an aqueous H_2_O_2_ solution (1 mM). The resulting suspension was then purged with argon gas for 30 min to remove dissolved oxygen. During the irradiation, 1 mL of suspension was collected every 15 min and filtered through a 0.22 μm membrane to remove the photocatalyst. The H_2_O_2_ concentration was determined using the iodometry method.

**3.4 Apparent quantum yield (AQY)**

The reaction conditions and methods are the same as those of photocatalytic hydrogen peroxide generation, except that an LED with a wavelength of 405 nm is used as light source. The AQY was calculated according to the following equation:

$$AQY\% =\frac{the number of evolved H_{2}O_{2} molecules \times2}{photon number} \times100\%$$

$$The number of evolved H_{2}O_{2} molecules = n \times N_{A}$$

$$N_{photon number} = \frac{qSt\lambda}{\mathrm{hc}}$$

where n is the molar mass of produced H_2_O_2_, N_A_ is Avogadro's constant (6.02×10^23^), h is Planck's constant (6.63×10^−34^ J s), c is the speed of light (3.0×10^8^ ms^−1^), q is the light intensity of incident light (11.668 mW cm^−2^), S is the illumination area (34.2 cm^2^), t is the time of photocatalytic reaction (10800 s), and λ is the wavelength of incident light (405 nm).

**3.5 Cycle experiment**

The reaction conditions were consistent with the photocatalytic H_2_O_2_ production tests. After 1 h of reaction, photocatalyst particles were collected, washed with water three times, and dried at 60 ºC. Then, it was re-dispersed in pure water for the next cycle.

**Figure S2.** FT-IR spectra of benzobisthiazole-based conjugated polymers.

**Figure S3.** (a) XPS survey spectra for BBTz-1D. High-resolution XPS spectra of (b) C 1s, (c) N 1s, and (d) S 2p for BBTz-1D, respectively.

**Figure S4.** (a) XPS survey spectra for BBTz-2D. High-resolution XPS spectra of (b) C 1s, (c) N 1s, and (d) S 2p for BBTz-2D, respectively.

**Figure S5.** (a) XPS survey spectra for BBTz-3D. High-resolution XPS spectra of (b) C 1s, (c) N 1s, and (d) S 2p for BBTz-3D, respectively.

**Figure S6.** Nitrogen adsorption/desorption isotherms and pore size distribution for (a) BBTz-1D, (b) BBTz-2D, and (c) BBTz-3D recorded at 77 K.

**Figure S7.** TGA curves of (a) BBTz-1D, (b) BBTz-2D, and (c) BBTz-3D.

**Figure S8.** XRD patterns of (a) BBTz-1D, (b) BBTz-2D, and (c) BBTz-3D.

**Figure S9.** SEM images of (a, b) BBTz-1D, (c, d) BBTz-2D, and (e, f) BBTz-3D.

**Figure S10.** (a, b) TEM images, (c) high–angle annular dark–field (HAADF) image, and (d-f) the corresponding EDS mapping of C (red), S (green), and N (blue) images of BBTz-1D.

**Figure S11.** (a, b) TEM images, (c) HAADF image, and (d-f) the corresponding EDS mapping of C (red), S (green), and N (blue) images of BBTz-2D.

**Figure S12.** (a, b) TEM images, (c) HAADF image, and (d-f) the corresponding EDS mapping of C (red), S (green), and N (blue) images of BBTz-3D.

**Figure S13.** Optical photograph of benzobisthiazole-based conjugated polymers powder.

**Figure S14.** UPS spectra of (a) BBTz-1D, (b) BBTz-2D, and (c) BBTz-3D.

**Figure S15.** Zeta potential values of benzobisthiazole-based conjugated polymers.

**Figure S16.** Electrochemical impedance spectroscopy **(**EIS) of BBTz-1D, BBTz-2D, and BBTz-3D.

**Figure S17.** EIS of (a) BBTz-1D, (b) BBTz-2D, and (c) BBTz-3D measured at different temperatures.

**Figure S18.** Transient photocurrent responses of BBTz-1D, BBTz-2D, and BBTz-3D.

**Figure S19.** (a) Steady-state PL spectra and (b) time-resolved PL decay curves of BBTz-1D, BBTz-2D, and BBTz-3D.

**Figure S20.** Time-resolved photocatalytic H_2_O_2_ production rates of BBTz-1D, BBTz-2D, and BBTz-3D in air atmospheres.

**Figure S21.** Dependence of AQY at 405 nm on catalyst loading for BBTz-3D.

**Figure S22.** Typical time course of photocatalytic H_2_O_2_ production over different catalysts in pure water under simulated white LED.

**Figure S23.** Long-term photocatalytic hydrogen peroxide production experiments were conducted under outdoor natural sunlight conditions using a self-assembled solar photoelectric conversion device.

**Figure S24.** Time course of photocatalytic H_2_O_2_ generation by BBTz-3D photocatalyst in (a) pure water (July 10 to 15, 2025) and (b) pure water: benzyl alcohol = 9:1 (v/v) (July 17 to 22, 2025).

**Figure S25.** (a) The cyclic plot of BBTz-1D, BBTz-2D and BBTz-3D photocatalytic hydrogen peroxide production efficiency under pure water. Cycling test of H_2_O_2_ production performance for (b) BBTz-1D, (c) BBTz-2D and (d) BBTz-3D in pure water and pure water: benzyl alcohol = 9:1 (v/v) under irradiation.

**Figure S26.** The XRD spectra of (a) BBTz-1D，(b) BBTz-2D and (c) BBTz-3D before and after photocatalytic reaction.

**Figure S27.** The FT-IR spectra of (a) BBTz-1D，(b) BBTz-2D and (c) BBTz-3D before and after photocatalytic reaction.

**Figure S28.** The highest occupied molecular orbital (HOMO) and the lowest unoccupied molecular orbital (LUMO) orbital distributions of the simplified benzobisthiazole-based conjugated polymers segments based on DFT calculation.

**Figure S29.** Comparison between the TD-DFT calculated singlet excited states (Structural model 1: BBTz-3D tetramer model; Structural model 2: BBTz-3D dimer model) and experimental absorption spectrum of BBTz-3D.

| **S_0_→S_1_** | |
| --- | --- |
| **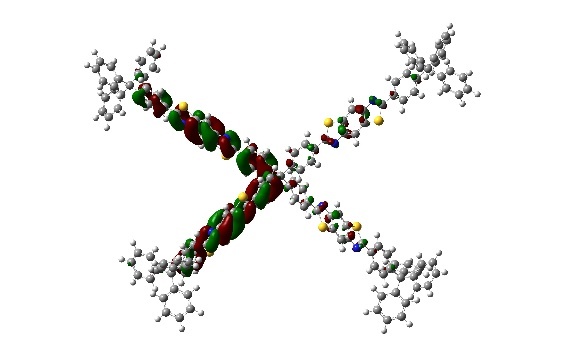** | **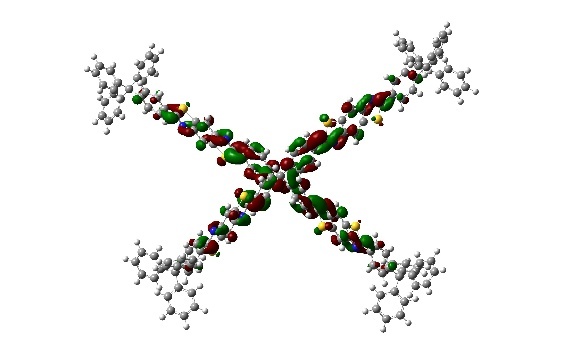** |
| **HOMO** | **LUMO** |
| **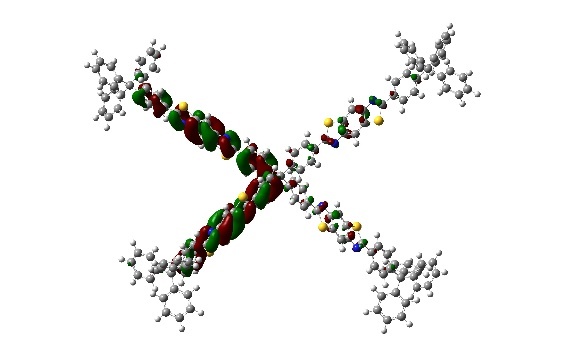** | **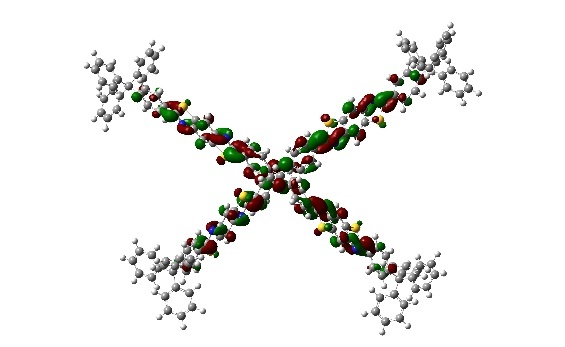** |
| **HOMO** | **LUMO+1** |
| **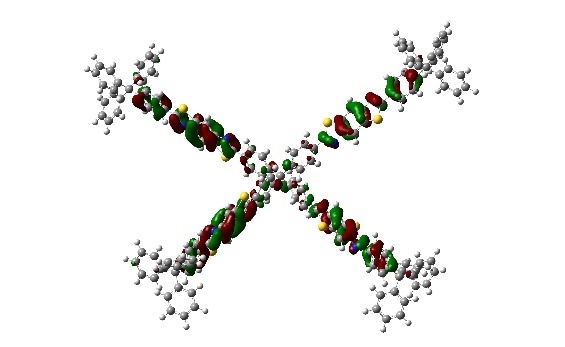** | **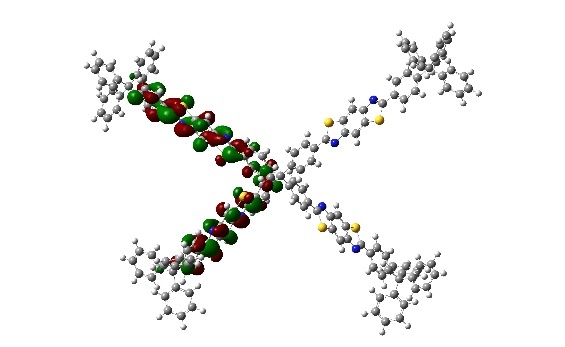** |
| **HOMO−2** | **LUMO+2** |
| **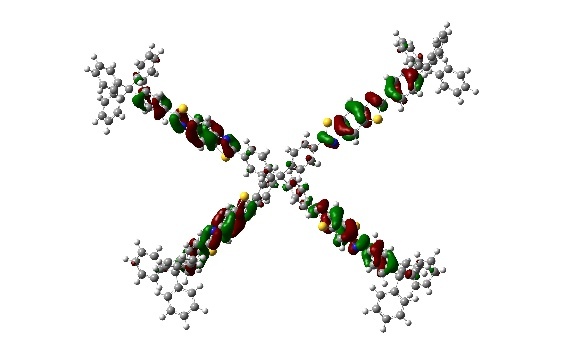** | **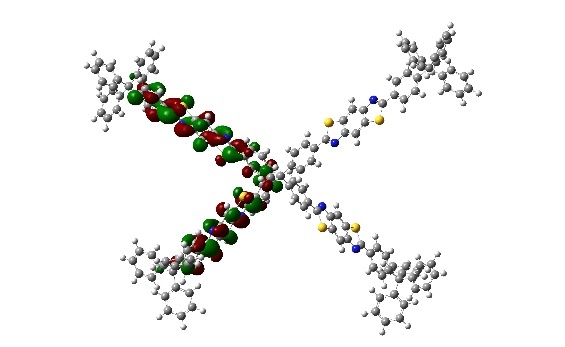** |
| **HOMO−3** | **LUMO+2** |

**Figure S30.** Frontier molecular orbital diagrams of BBTz-3D tetramer model, depicting the HOMO and LUMO distributions for the S_0_→S_1_ transition.

| **S_0_→S_2_** | |
| --- | --- |
| **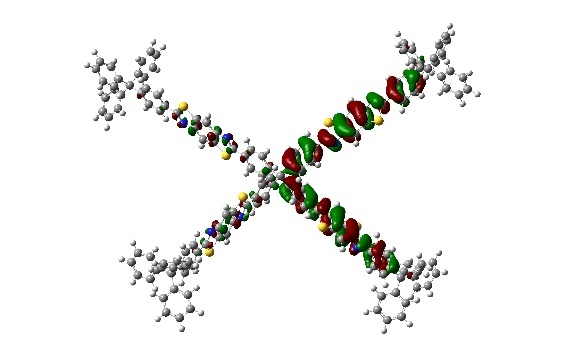** | **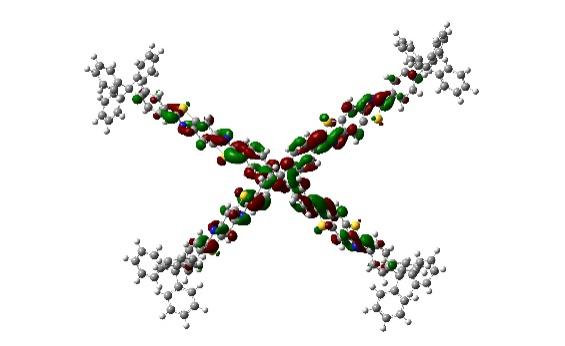** |
| **HOMO−1** | **LUMO** |
| **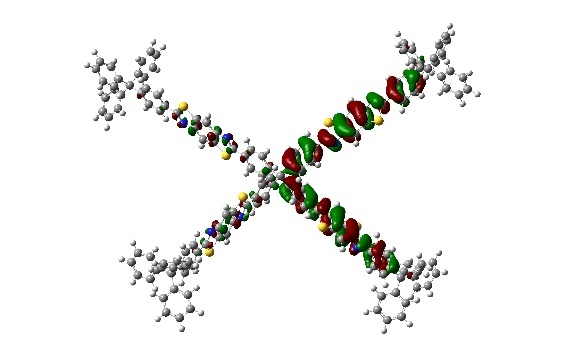** | **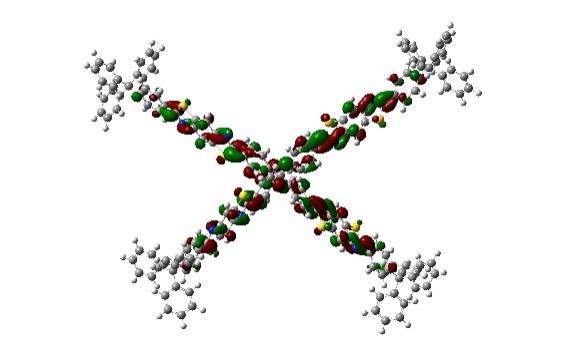** |
| **HOMO−1** | **LUMO+1** |
| **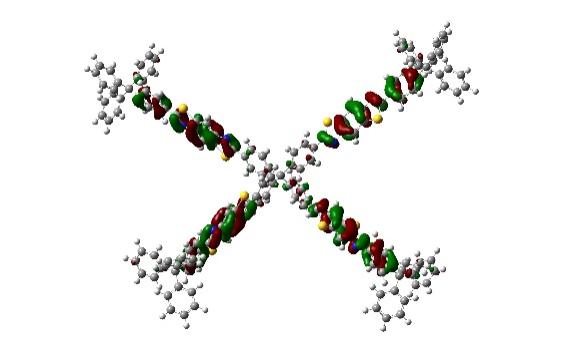** | **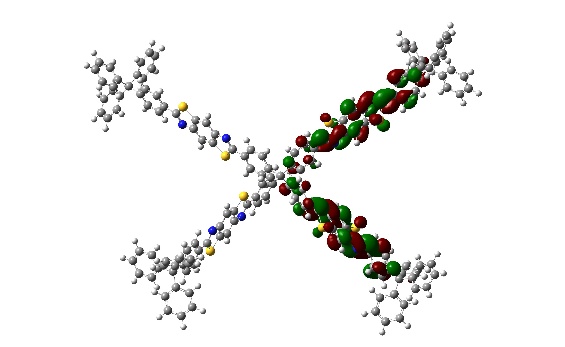** |
| **HOMO−3** | **LUMO+3** |
| **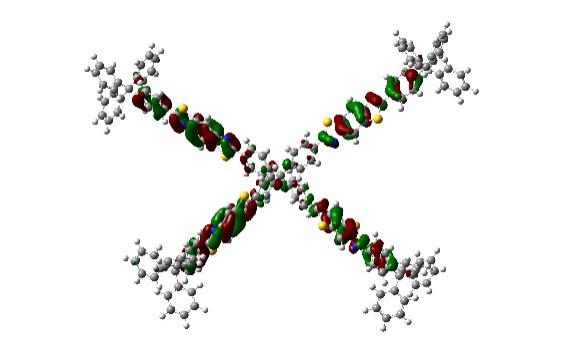** | **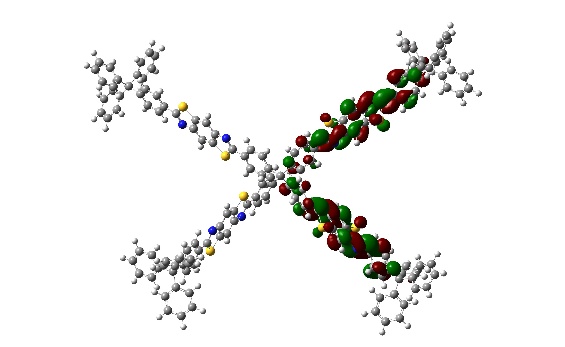** |
| **HOMO−2** | **LUMO+3** |

**Figure S31.** Frontier molecular orbital diagrams of BBTz-3D tetramer model, depicting the HOMO and LUMO distributions for the S_0_→S_2_ transition.

| **S_0_→S_3_** | |
| --- | --- |
| **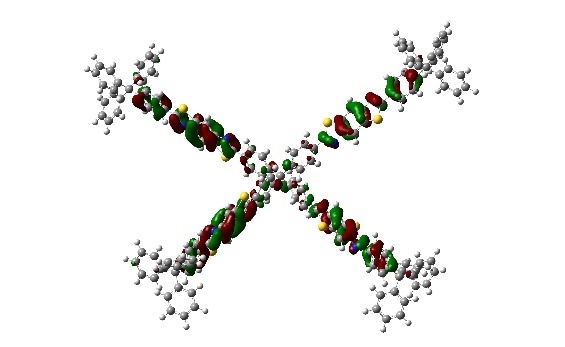** | **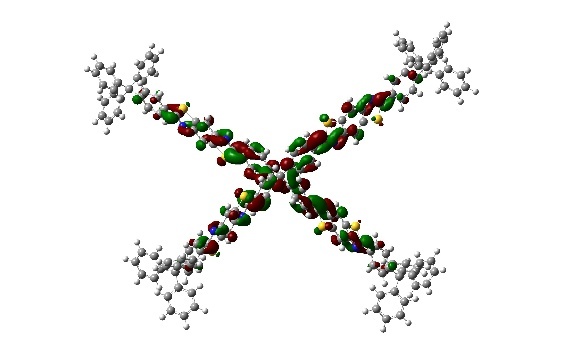** |
| **HOMO−2** | **LUMO** |
| **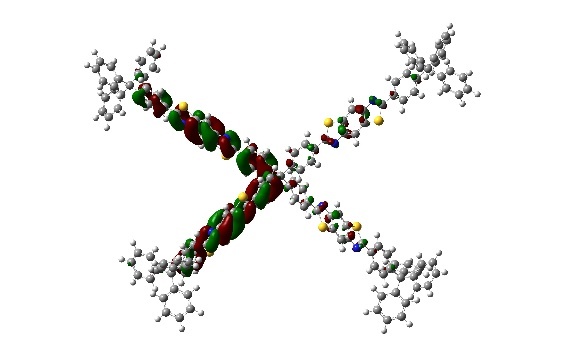** | **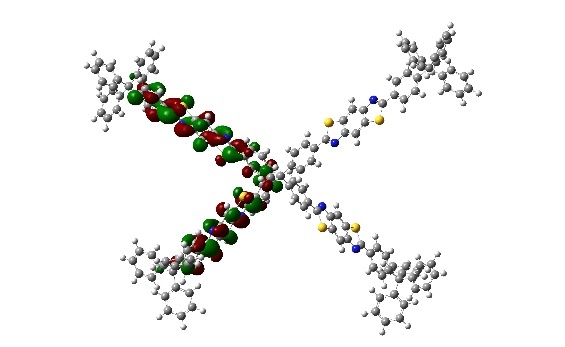** |
| **HOMO** | **LUMO+2** |
| **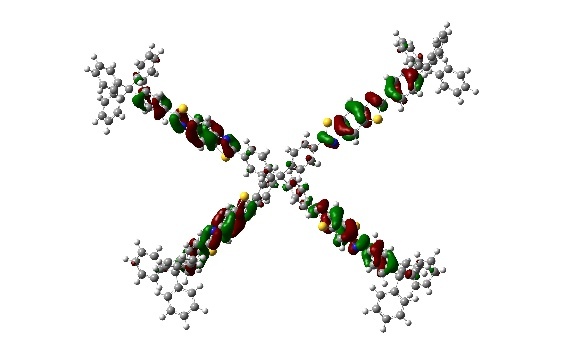** | **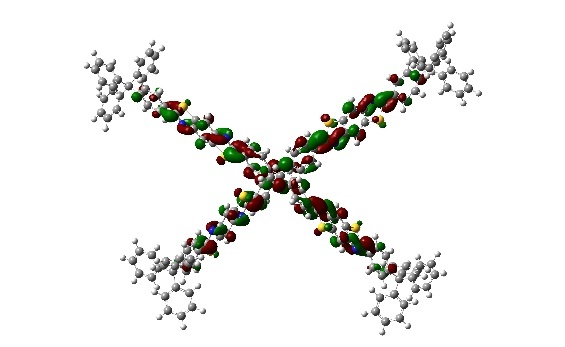** |
| **HOMO−3** | **LUMO+1** |
| **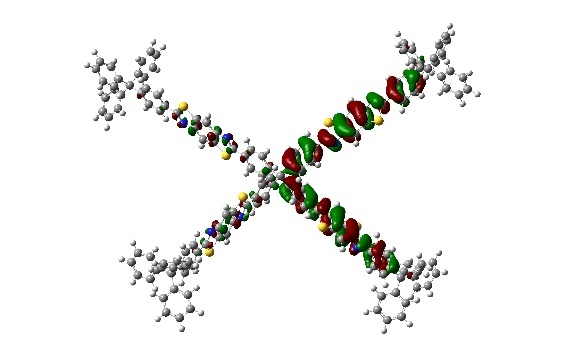** | **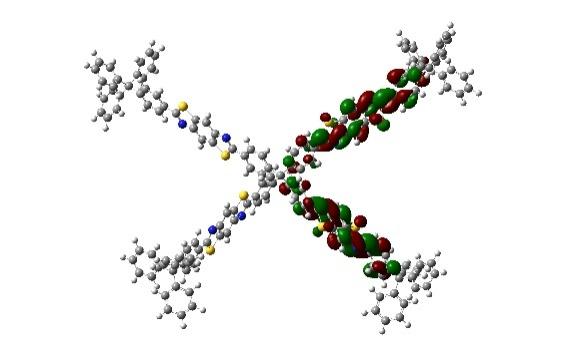** |
| **HOMO−1** | **LUMO+3** |

**Figure S32.** Frontier molecular orbital diagrams of BBTz-3D tetramer model, depicting the HOMO and LUMO distributions for the S_0_→S_3_ transition.

| **S_0_→S_1_** | |
| --- | --- |
| **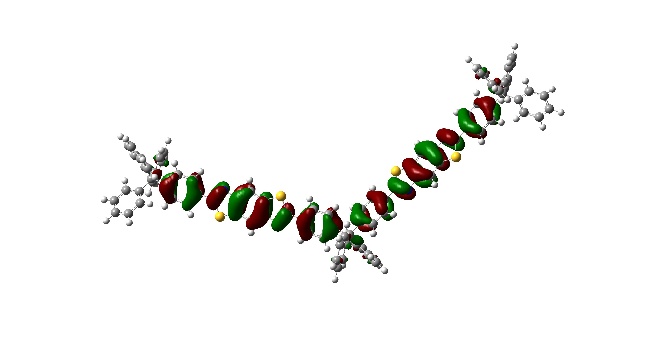** | **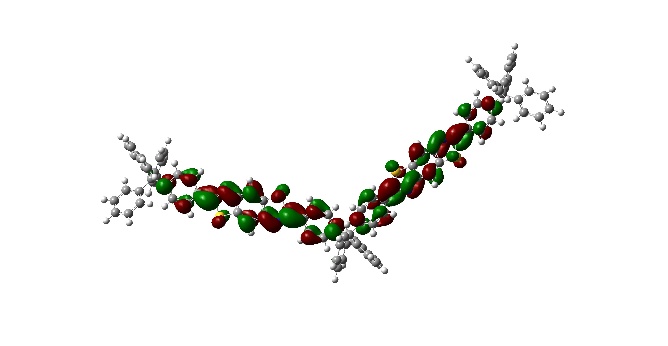** |
| **HOMO** | **LUMO** |
| **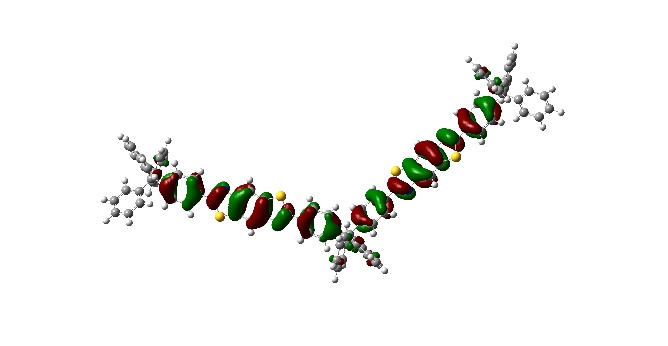** | **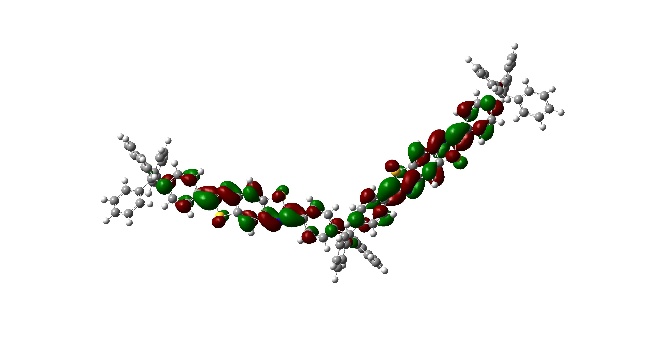** |
| **HOMO−1** | **LUMO+1** |

**Figure S33.** Frontier molecular orbital diagrams of BBTz-3D dimer model, depicting the HOMO and LUMO distributions for the S_0_→S_1_ transition.

| **S_0_→S_2_** | |
| --- | --- |
| **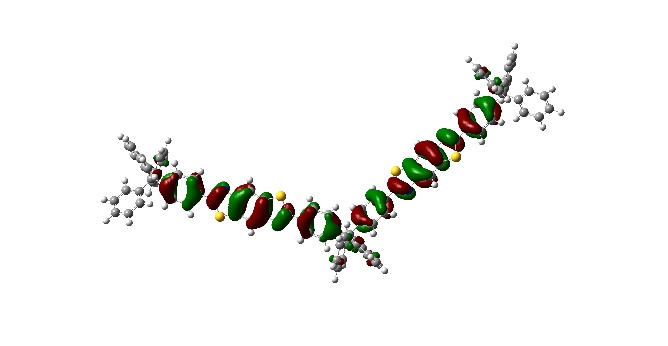** | **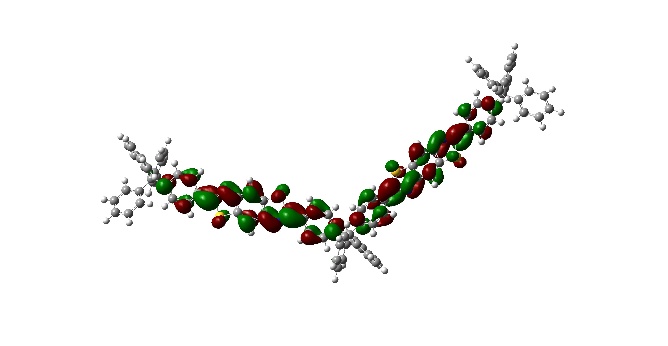** |
| **HOMO−1** | **LUMO** |
| **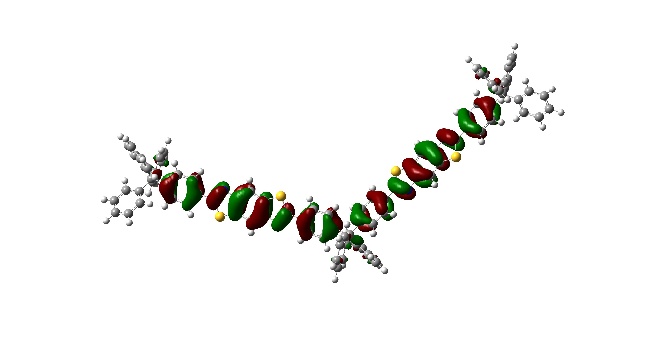** | **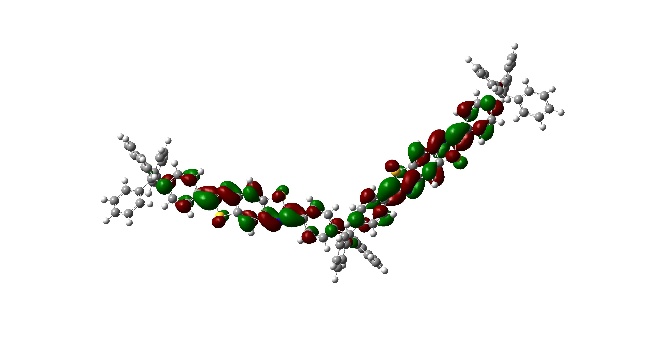** |
| **HOMO** | **LUMO+1** |

**Figure S34.** Frontier molecular orbital diagrams of BBTz-3D dimer model, depicting the HOMO and LUMO distributions for the S_0_→S_2_ transition.

| **S_0_→S_3_** | |
| --- | --- |
| **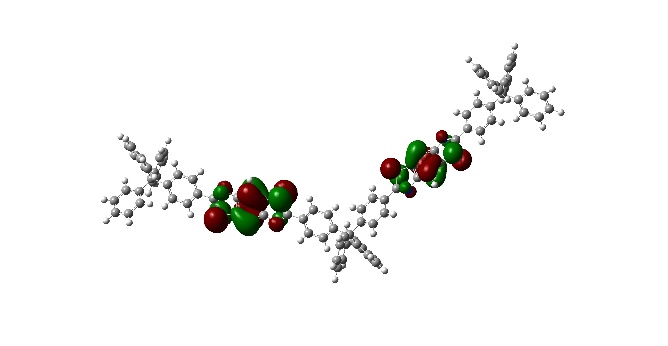** | **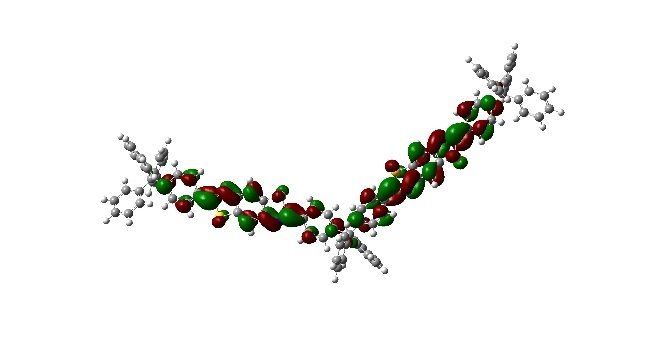** |
| **HOMO−2** | **LUMO+1** |
| **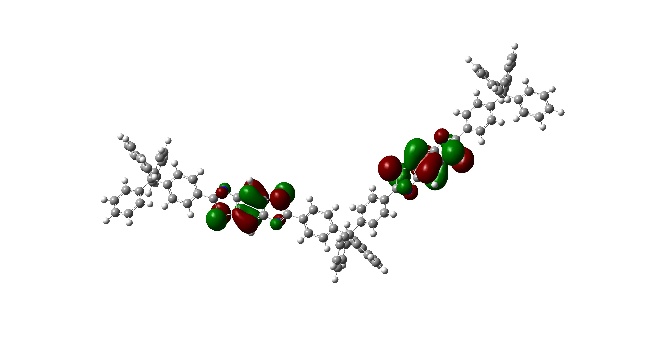** | **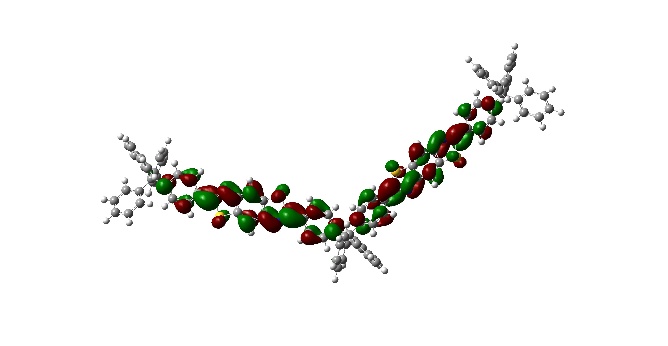** |
| **HOMO−3** | **LUMO** |
| **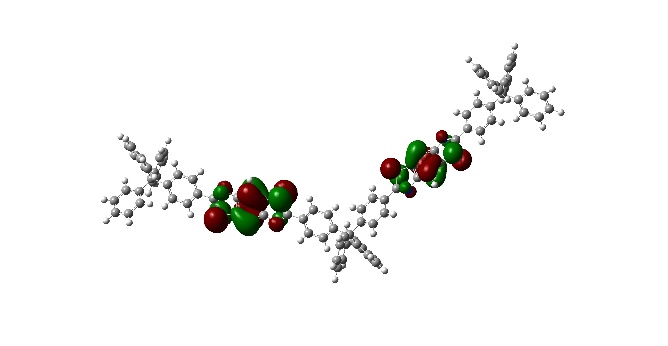** | **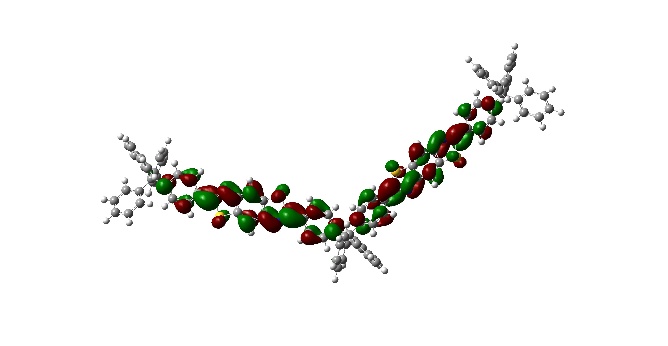** |
| **HOMO−2** | **LUMO** |

**Figure S35.** Frontier molecular orbital diagrams of BBTz-3D dimer model, depicting the HOMO and LUMO distributions for the S_0_→S_3_ transition.

**Table S1.** Fitted parameters and average lifetimes (τ_ave_) of fluorescence lifetime decay spectra.

| **Catalysts** | **A_1_** | **τ_1_(ns)** | **A_2_** | **τ_2_(ns)** | **τ_ave_*(ns)** |
| --- | --- | --- | --- | --- | --- |
| BBTz-1D | 0.85 | 1.69 | 0.13 | 8.42 | 4.60 |
| BBTz-2D | 0.84 | 1.83 | 0.19 | 8.07 | 4.95 |
| BBTz-3D | 0.86 | 2.31 | 0.15 | 10.66 | 6.03 |

$$*\tau_{\mathbf{ave}}=(A_{1}\times\tau_{1}^{2}+A_{2}\times\tau_{2}^{2})/(A_{1}\times\tau_{1}+A_{2}\times\tau_{2})$$

**Table S2.** Photocatalyst for H_2_O_2_ production under natural sunlight conditions.

| **Photocatalyst** | **Conditions** | | **H_2_O_2_** | **Ref.** |
| --- | --- | --- | --- | --- |
|  | **Solvent** | **Gas** |  |  |
| BBTz-1D | lake water  (Erhai Lake) | Air | 4989.28  (μmol g^−1^) | This work |
| BBTz-2D |  |  | 6104.56  (μmol g^−1^) |  |
| BBTz-3D |  |  | 6737.27  (μmol g^−1^) |  |
| BBTz-3D | pure water | Air | 10.04  (mmol g^−1^) (Cumulative H_2_O_2_ production was measured from samples collected daily at 9:00, 15:00, and 21:00) |  |
|  | pure water : benzyl alcohol = 9:1 | Air | 744.97  (mmol g^−1^) (Cumulative H_2_O_2_ production was measured from samples collected daily at 9:00, 15:00, and 21:00) |  |
| DMCR-1NH | seawater: benzyl alcohol = 10:1 | O_2_ | 1319  (μmol g^−1^ h^−1^) | ^[16]^ |
| PMCR-1 | seawater: benzyl alcohol = 10:1 | O_2_ | 2736  (μmol g^−1^ h^−1^) | ^[17]^ |
| O-PTAQ | pure water | Air | 4989  (μmol g^−1^ h^−1^) | ^[18]^ |
| COF-2CN | pure water | Air | 1368  (μmol g^−1^ h^−1^) | ^[19]^ |
| COF-C | pure water | Air | 574  (μmol h^−1^) | ^[20]^ |
|  | seawater |  | 387  (μmol h^−1^) |  |
| COF-TPDB-NO_2_ | water | Air | 4.14  (mM m^−2^ day^−1^) (calculated based on an irradiation time of 8 h per day) | ^[21]^ |
| B[f]QCOF-1 | pure water | O_2_ | 5060  (μmol g^−1^ h^−1^) | ^[22]^ |
|  | seawater |  | 4365  (μmol g^−1^ h^−1^) |  |
| COF-N32 | ultrapure water | Air | 602  (μmol g^−1^ h^−1^) | ^[23]^ |
|  | river water |  | 515  (μmol g^−1^ h^−1^) |  |
|  | tap water |  | 475  (μmol g^−1^ h^−1^) |  |
|  | seawater |  | 396  (μmol g^−1^ h^−1^) |  |
| SA-SADF-H^+^ | water | Air | 2815.7  (μmol L^−1^ h^−1^) | ^[24]^ |
| Kf-F-COF | water | O_2_ | 4195.56  (μmol g^−1^ h^−1^) | ^[25]^ |
| CityU-41 | distilled water | Air | 534.1  (μmol g^−1^ h^−1^) | ^[26]^ |

**Table S3.** Various photocatalysts for H_2_O_2_ production.

| **Photocatalyst** | **Conditions** | | **H_2_O_2_**  **(μmol g^−1^ h^−1^)** | **Ref.** |
| --- | --- | --- | --- | --- |
|  | **Solvent** | **Gas** |  |  |
| BBTz-1D | H_2_O | Air | 5367.29 | This  work |
|  |  | O_2_ | 7196.60 |  |
| BBTz-2D |  | Air | 6932.98 |  |
|  |  | O_2_ | 7816.80 |  |
| BBTz-3D |  | Air | 7970.51 |  |
|  |  | O_2_ | 8315.46 |  |
| BBTz | H_2_O | Air | 7274 | ^[27]^ |
| Ni_1_Zn_1_-PCN | H_2_O | O_2_ | 1205.4 | ^[28]^ |
| BTT-H3 COF | H_2_O | Air | 1588 | ^[29]^ |
| Ald-TTB-TTA | H_2_O | O_2_ | 3169 | ^[30]^ |
| EBBT-COF | H_2_O | O_2_ | 5686 | ^[31]^ |
| PTTN-AO | H_2_O | O_2_ | 6024 | ^[32]^ |
| TMBCOF | H_2_O | Air | 9613 | ^[33]^ |
|  |  | O_2_ | 10762 |  |
| TP-DPBD_30_-COF | H_2_O | Air | 7200 | ^[34]^ |
| CZ-AQ | H_2_O | Air | 4401 | ^[35]^ |
| QCD^36^A_1-1_350 | H_2_O | Air | 2510 | ^[36]^ |
| TTB-TTA-Ph-3F | H_2_O | Air | 3496.9 | ^[37]^ |
| TPB-COF-OH | H_2_O | Air | 6608 | ^[38]^ |
| Hf-PMOF/APF(HA-2) | H_2_O | O_2_ | 2995.13 | ^[39]^ |
| BD-CQD | H_2_O | Air | 1562 | ^[40]^ |
| TaptBtt | H_2_O | O_2_ | 1407 | ^[41]^ |
| PABA/CN | H_2_O | O_2_ | 745 | ^[42]^ |
| 10 wt%-PANI/CdS-NP | H_2_O | Air | 371.33 | ^[43]^ |
| B[f]QCOF-1 | H_2_O | O_2_ | 9025 | ^[22]^ |
| PAF-379 | H_2_O | Air | 7124 | ^[44]^ |
| Co@rGO-P/PTA | H_2_O | O_2_ | 1400 | ^[45]^ |
| UIO-66-$\mathrm{NH}_{2}^{\mathrm{etch}}$@His-SOD | H_2_O | Air | 3890.37 | ^[46]^ |
| iTPPy-COF | H_2_O | Air | 6249 | ^[47]^ |
|  |  | O_2_ | 7955 |  |
| COF-BTT-TAPT | H_2_O | O_2_ | 620 | ^[48]^ |
| BYTT-COF | H_2_O | Air | 8051 | ^[49]^ |
|  |  | O_2_ | 9461 |  |
| USTB-10-S | H_2_O | O_2_ | 5041 | ^[50]^ |
| HHT-COF | H_2_O | Air | 4996 | ^[51]^ |
| PTTN-AO | H_2_O | O_2_ | 6024 | ^[52]^ |
| SZ | H_2_O | O_2_ | 3109 | ^[53]^ |
| DMAP-BNCOFs | H_2_O | O_2_ | 8051.4 | ^[54]^ |

**References**

[1] G. Kresse, J. Furthmüller, *Comput. Mater. Sci.* **1996**, 6, 15.

[2] P. E. Blochl, O. Jepsen, O. K. Andersen, *Phys. Rev. B.* **1994**, 49, 16223.

[3] J. P. Perdew, J. A. Chevary, S. H. Vosko, K. A. Jackson, M. R. Pederson, D. J. Singh, C. Fiolhais, *Phys. Rev. B.* **1993**, 48, 4978.

[4] S. Grimme, J. Antony, S. Ehrlich, H. Krieg, *J. Chem. Phys.* **2010**, 132, 154104.

[5] F. Weigend, *Phys Chem Chem Phys*. **2006**, 8, 1057.

[6] F. Weigend, R. Ahlrichs, *Phys. Chem. Chem. Phys.* **2005**, 7, 3297.

[7] Y. Zhao, D. G. Truhlar, *Theor. Chem. Acc.* **2007**, 120, 215.

[8] A. V. Marenich, C. J. Cramer, D. G. Truhlar, *J. Phys. Chem. B* **2009**, 113, 6378.

[9] S. Miertuš, E. Scrocco, J. Tomasi, *Chem. Phys.* **1981**, 55, 117.

[10] S. Miertus̃, J. Tomasi, *Chem. Phys.* **1982**, 65, 239.

[11] M. Frisch, G. Trucks, H. Schlegel, G. Scuseria, M. Robb, J. Cheeseman, G. Scalmani, V. Barone, G. Petersson, H. Nakatsuji, **2016**, http://gaussian.com/citation/.

[12] T. Lu, *J. Chem. Phys.* **2024**, 161, 082503.

[13] Z. Liu, T. Lu, Q. Chen, *Carbon*. **2020**, 165, 461.

[14] T. Lu, F. Chen, *J. Comput. Chem.* **2012**, 33, 580.

[15] W. Humphrey, A. Dalke, K. Schulten, *J. Mol. Graph.* **1996**, 14, 33.

[16] P. Das, G. Chakraborty, J. Roeser, S. Vogl, J. Rabeah, A. Thomas, *J. Am. Chem. Soc.* **2023**, 145, 2975.

[17] P. Das, J. Roeser, A. Thomas, *Angew. Chem. Int. Ed.* **2023**, 62, 202304349.

[18] P. Jiang, Y. Huang, X. Jiang, H. Yan, S. Liu, Z. Chen, X. Wu, X. Zhou, Y. X. Ye, G. Ouyang, *Adv. Sci.* **2025**, 12, 03929.

[19] Y. Hou, P. Zhou, F. Liu, Y. Lu, H. Tan, Z. Li, M. Tong, J. Ni, *Angew. Chem. Int. Ed.* **2024**, 63, 202318562.

[20] S. Maity, G. K. Dam, S. Rasaily, A. Roy, S. K. Ghosh, *ChemRxiv.* **2024**, https://doi.org/ 10.26434/chemrxiv-2024-bqf8n.

[21] J. Su, B. Liu, B. Lu, X. Sun, Y. Guo, W. Chi, Y. Yang, X. Chen, H. Zhao, Y. Wang, H. Miao, H. Zhu, Y. Dong, Y. Zhu, *Appl. Catal. B: Environ. Energy* **2025**, 371, 125263.

[22] K.-H. Xie, G.-B. Wang, F. Huang, F. Zhao, J.-L. Kan, Z.-Z. Chen, L. Cai, S.-L. Han, Y. Geng, Y.-B. Dong, *Nat. Commun.* **2025**, 16, 3493.

[23] F. Liu, P. Zhou, Y. Hou, H. Tan, Y. Liang, J. Liang, Q. Zhang, S. Guo, M. Tong, J. Ni, *Nat. Commun.* **2023**, 14, 4344.

[24] W. Li, B. Han, Y. Liu, J. Xu, H. He, G. Wang, J. Li, Y. Zhai, X. Zhu, Y. Zhu, *Angew. Chem. Int. Ed.* **2024**, 64, 202421356.

[25] W. Tao, Y. Wang, L. Cong, C. Zhang, Y. Gao, H. Zheng, W. Shi, D. Zhong, T. Lu, *Sci. China Chem.* **2025**, https://doi.org/10.1007/s11426-025-2840-0.

[26] L. Zhang, Z. Chen, X.-X. Li, X. Wang, Q. Gu, Z. Zheng, N. Aratani, C.-S. Lee, Y.-Q. Lan, Q. Zhang, *J. Am. Chem. Soc.* **2025**, 147, 27847.

[27] J. Cheng, S. Wan, S. Cao, *Angew. Chem. Int. Ed.* **2023**, 62, 202310476.

[28] J.-R. Huang, H.-Y. Chen, H.-L. Zhu, P.-Q. Liao, X.-M. Chen, *J. Am. Chem. Soc.* **2025**, 147, 37167.

[29] A. Chakraborty, A. Alam, U. Pal, A. Sinha, S. Das, T. Saha-Dasgupta, P. Pachfule, *Nat. Commun.* **2025**, 16, 503.

[30] Q. Rong, X. Chen, Z. Huang, S. Li, S. He, *ACS Appl. Mater. Interfaces.* **2025**, 17, 3163.

[31] B. Li, J. Chen, K. Wang, D. Qi, T. Wang, J. Jiang, *Adv. Energy Mater.* **2025**, 15, 2404497.

[32] Z. Yu, F. Yu, M. Xu, S. Feng, J. Qiu, J. Hua, *Adv. Sci.* **2025**, 12, 2415194.

[33] J. Zhang, F. Xue, Z. Wang, *Small*. **2025**, 21, 07052.

[34] Y. Chen, R. Liu, Y. Guo, G. Wu, T. C. Sum, S. W. Yang, D. Jiang, *Nat. Synth.* **2024**, 3, 998.

[35] H. Yan, J. Jiang, Y. Huang, M. Shen, J. Xu, Y. X. Ye, G. Ouyang, *Adv. Mater.* **2025**, 07961, https://doi.org/10.1002/adma.202507961.

[36] B. Jiang, D. Chen, N. Li, Q. Xu, H. Li, J. Lu, *Chem. Eng. J.* **2024**, 487, 150609.

[37] Q. Rong, X. Chen, Q. Cheng, Z. Huang, S. He, *ACS Sustain. Chem. Eng.* **2024**, 12, 13306.

[38] S. Feng, H. Cheng, F. Chen, X. Liu, Z. Wang, H. Xu, J. Hua, *ACS Catal*. **2024**, 14, 7736.

[39] H. He, Z. Wang, J. Zhang, S. Mamatkulov, O. Ruzimuradov, K. Dai, J. Low, Y. Li, *Energy Environ. Sci.* **2025**, 18, 6191.

[40] X. Zhang, L. Ma, S. Ding, N. Meng, W. Wang, *ACS Nano*. **2025**, 19, 24078.

[41] C. Qin, X. Wu, L. Tang, X. Chen, M. Li, Y. Mou, B. Su, S. Wang, C. Feng, J. Liu, X. Yuan, Y. Zhao, H. Wang, *Nat. Commun*. **2023**, 14, 5238.

[42] M. He, X. Peng, S. Wu, B. Lei, S. Xiong, Q. Luo, Z. Tu, X. Lin, G. Peng, *Inorg. Chem. Front.* **2025**, 12, 3237.

[43] H. Wang, Q. Zhang, Y. Yang, J. Bian, C. Li, *J. Environ. Chem. Eng.* **2024**, 12, 114979.

[44] H. Wang, L. Cao, X. Tao, G. Zhu, *Angew. Chem. Int. Ed.* **2025**, 64, 202502943.

[45] Q. Wang, L. Meng, Z. Li, Z. Yang, Y. Tang, L. Yu, Z. Li, J. Sun, L. Jing, *Chin. J. Catal.* **2025**, 75, 192.

[46] X. Ma, J. Han, J. Wu, Y. Zhou, C. Li, L. Wang, Y. Lu, Y. Wang, *Int. J. Biol. Macromol.* **2025**, 326, 147260.

[47] J. Zhang, F. Xue, Z. Wang, *Angew. Chem. Int. Ed.* **2025**, 64, 202425617.

[48] M. Liu, P. He, H. Gong, Z. Zhao, Y. Li, K. Zhou, Y. Lin, J. Li, Z. Bao, Q. Yang, Y. Yang, Q. Ren, Z. Zhang, *Chem Eng J.* **2024**, 482, 148922.

[49] J.-Y. Yue, Z.-S. Xu, J.-X. Luo, P. Yang, B. Tang, *ACS Catal.* **2025**, 15, 12541.

[50] X. Ding, T. Wang, B. Yu, Q. Zhi, H. Wang, H. Liu, P. A. Stuzhin, J. Jiang, *Adv. Funct. Mater.* **2025**, 35, 2422291.

[51] Z. X. Pan, Y. Guo, J. X. Luo, J. Y. Yue, *Adv. Funct. Mater.* **2025**, 12475, https://doi.org/10.1002/adfm.202512475.

[52] Z. Yu, F. Yu, M. Xu, S. Feng, J. Qiu, J. Hua, *Adv. Sci.* **2025**, 12, 2415194.

[53] T. Li, Y. Wang, H. Shen, H. Zhu, R. Wang, S. Wu, C. Jin, L. Chen, Y. Li, X. Yan, B. Wang, *Appl. Catal. A.* **2025**, 703, 120338.

[54] M. Chen, J. Wang, T. Wei, B. Zhang, K. Guo, Y. Feng, B. Zhang, *J. Am. Chem. Soc.* **2025**, 147, 24050.
